# *FABP4* and *MMP9* levels identified as predictive factors for poor prognosis in patients with nonalcoholic fatty liver using data mining approaches and gene expression analysis

**DOI:** 10.1038/s41598-019-56235-y

**Published:** 2019-12-24

**Authors:** Audrey Coilly, Christophe Desterke, Catherine Guettier, Didier Samuel, Franck Chiappini

**Affiliations:** 1 Inserm, UMR-U1193, Villejuif, F-94800 France; 2 Univ Paris-Sud, Institut André Lwoff, Villejuif, F-94800 France; 3 DHU Hepatinov, Villejuif, F-94800 France; 4 AP-HP, Centre Hépatobiliaire, Hôpital Paul Brousse, Villejuif, F-94800 France; 5 Inserm, UMR-935, Villejuif, F-94800 France; 6 AP-HP, Service d’Anatomopathologie, Hôpital Bicêtre, Le Kremlin-Bicêtre, F-94275 France; 7 Laboratoire Croissance, Régénération, Réparation et Régénération Tissulaires (CRRET)/EAC CNRS 7149, Univ Paris-Est Créteil (UPEC), F-94010 Créteil, France

**Keywords:** Microarrays, Hepatology

## Abstract

Nonalcoholic fatty liver (NAFLD) may progress to nonalcoholic steatohepatitis (NASH) and ultimately to cirrhosis and hepatocellular carcinoma (HCC). Prognostic markers for these conditions are poorly defined. The aim of this study was to identify predictive gene markers for the transition from NAFL to NASH and then to poorer conditions. Gene expression omnibus datasets associated with a prediction analysis algorithm were used to create a matrix composed of control subject (n = 52), healthy obese (n = 51), obese with NAFL (n = 42) and NASH patients (n = 37) and 19,085 genes in order to identify specific genes predictive of the transition from steatosis to NASH and from NASH to cirrhosis and HCC and thus patients at high risk of complications. A validation cohort was used to validate these results. We identified two genes, fatty acid binding protein-4 (FABP4) and matrix metalloproteinase-9 (MMP9), which respectively allowed distinguishing patients at risk of progression from NAFL to NASH and from NASH to cirrhosis and HCC. Thus, NAFL patients expressing high hepatic levels of FABP4 and NASH patients expressing high hepatic levels of MMP9 are likely to experience disease progression. Therefore, using FABP4 and MMP9 as blood markers could help to predict poor outcomes and/or progression of NAFL during clinical trial follow-up.

## Introduction

Nonalcoholic fatty liver disease (NAFLD) includes a wide spectrum of conditions from nonalcoholic fatty liver (NAFL) or simple steatosis to nonalcoholic steatohepatitis (NASH) which may progress to hepatic fibrosis, cirrhosis and ultimately to hepatocellular carcinoma (HCC)^1–5^. Indeed, 25–30% of NAFL patients will develop liver inflammation and progress to NASH and more than 30% of NASH patients will develop severe fibrosis and/or cirrhosis leading to HCC^1,6,7^. NASH is a liver disorder associated with obesity, insulin resistance, type 2 diabetes mellitus (T2D) and metabolic syndrome^6–9^. The incidence of NASH has dramatically increased and it is now the leading cause of chronic liver disease and a major public health issue worldwide^10–14^. In addition, NASH patients are at higher risk of cardiovascular diseases, which are also the leading cause of mortality in these patients^6^. Therefore, NAFL and NASH should be diagnosed at an early stage during which they may be reversible using lifestyle changes and/or a pharmacological management^15,16^.

Guidelines have been recently proposed to determine which patients should be screened for NAFLD such as patients with obesity, T2D and/or metabolic syndrome^17,18^. Non-invasive approaches have been proposed to differentiate NAFL from NASH such as the use of ultrasound or a fatty liver index (FLI) which includes the body mass index (BMI), waist circumference, triglyceride levels and serum gamma-glutamyl transferase (γ-GT) levels and to identify liver fibrosis with numerous non-invasive diagnostic tests^19^. In parallel, the primary and secondary causes of steatosis such as viral hepatitis C, autoimmune hepatitis, genetic mutations or polymorphisms, alcohol consumption, medications, total parenteral nutrition, congenital or acquired lipodystrophy, have to be ruled out. Based on the poor or contradictory results of these non-invasive approaches, or in the context of clinical trials, liver biopsy is highly recommended^16–18^.

Thus, despite the procedure-related risks of morbidity and mortality, liver biopsy remains the “gold standard” for the diagnosis of NASH. The NASH Clinical Research Network has developed a NAFLD activity score (NAS) based on steatosis grading, the presence of hepatocellular ballooning and lobular inflammation. A NAS below 3 indicates the absence of NASH while a NAS upper 5 supports the diagnosis of NASH. In addition, a fibrosis score may be associated with the NAS^16,20,21^. The diagnosis of a patient with a NAS ranging between 3 and 5 being unclear, this score is mainly used to assess NASH progression in clinical trials, but it cannot really be used as a diagnostic tool to identify NASH patients^16^. Recently, the “Fatty Liver Inhibition of Progression” (FLIP) European Consortium has focused on a diagnostic algorithm for NASH identification (presence of steatosis > 5%, hepatocellular ballooning and lobular inflammation) and has proposed a more accurate and reproducible score separating Steatosis, Activity and Fibrosis (SAF)^22,23^. However, the histological assessment of NAFLD patients remains strongly observer-dependent and is not fully reproducible^24,25^. In addition, it has also been shown that sampling variabilities (*e.g*. right *vs*. left lobe, needle size) may interfere with the diagnosis of NAFLD^24,26–28^. Therefore, the pathologist assessment of NAFLD lesions may be challenging^25,27,29,30^ and it may be assumed that the prevalence of NASH and its complications is probably underestimated^1,3,5,10,11,14,19,31^. Based on these observations, we have previously shown the need to confirm the pathological assessment with an analysis of gene expression levels and that genes involved in inflammatory processes are upregulated in patients with steatosis using high-density oligonucleotide microarrays^32^.

Despite many years of research to identify non-invasive predictive markers for NASH using sophisticated algorithm approaches, none of the known markers is reliable enough to remove the need to perform liver biopsy due to the large spectrum of disease manifestations ranging from simple steatosis to HCC^8,21,24,25,27,28,30^.

Because gene expression is highly specific and very sensitive to environmental changes, exploring comprehensive gene expression levels in liver biopsies may help to identify markers of progression from steatosis to NASH. Thus, the aim of this study was to identify predictive gene markers for the transition from NAFLD to NASH and then to poorer outcomes using hepatic gene expression microarray datasets.

## Methods

### Learning datasets and statistical analysis

GSE48452 and GSE61260 microarray datasets were selected and downloaded from the genome expression omnibus (GEO) database on NCBI website (http://www.ncbi.nlm.nih.gov/geo/) and were used as learning datasets. Briefly, gene expression levels were measured in human liver biopsies using the same high-density oligonucleotide microarray ([HuGene-1_1-st] Affymetrix® Human Gene 1.1 ST Array [transcript (gene) version]) as previously published. Each array included more than 750,000 unique 25-mer oligonucleotide probes interrogating more than 28,000 genes^33,34^. GSE48452 and GSE61260 respectively included a total of 73 and 109 human liver samples grouped into control samples (n = 14 and 38), healthy obese (HO) samples (n = 27 and 24), obese and NAFL samples (n = 14 and 23) and obese and NASH samples (n = 18 and 24). Thus, a total of 52 control samples, 51 HO samples, 37 obese and NAFL samples and 42 obese and NASH samples could be analyzed when both GSE datasets were combined^33,34^. The characteristics of the patients who provided these samples are summarized in Table 1.

**Table 1:** Characteristics of patients from the learning dataset (GSE48452 and GSE61260).

Patients	Control N = 52	Healthy Obese N = 51	NAFL N = 37	NASH N = 42
Gender (F/M)	31/21	44/7	21/16	26/16
Age (year)	56.4 ± 18.2	47.1 ± 9.5**	41.4 ± 8.3**	45.3 ± 10.8**
BMI (kg/m^2^)	24.2 ± 3.2	41.7 ± 8.0***	41.4 ± 8.4***	49.0 ± 11.9***
Fat (area in %)	0 [0–1]	1 [0–3]	30 [20–52.5]	75 [70–80]
Inflammation	0 [0–0]	0 [0–0]	0 [0–0]	2 [1–2]
NAS	0 [0–0]	0 [0–0]	1.5 [1–2]	5 [4–6]
Fibrosis	0 [0–1]	0 [0–0]	0 [0–1]	1 [0–1]
Leptin level (pg/ml serum)	6.8 ± 6.4	29.1 ± 17.6***	43.4 ± 18.8***	35.7 ± 24.3***
Adiponectin level (pg/ml serum)	12.5 ± 9.6	8.3 ± 3.3	5.8 ± 1.6*	6.9 ± 3.1*

Data are expressed as a mean ± SEM or a median [interquartile]. Data were collected from previously published GSE48542 and GSE61260 datasets^33,34^. **p-value <0.01 and ***p-value <0.001 *versus* Controls (unpaired *t*-test). Gender distribution across the 4 groups: p-value <0.0001 (Chi-squared test). F: female; M: male; NAFL, nonalcoholic fatty liver; NAS: NAFLD activity score; NASH, nonalcoholic steatohepatitis.

**Figure 1:**
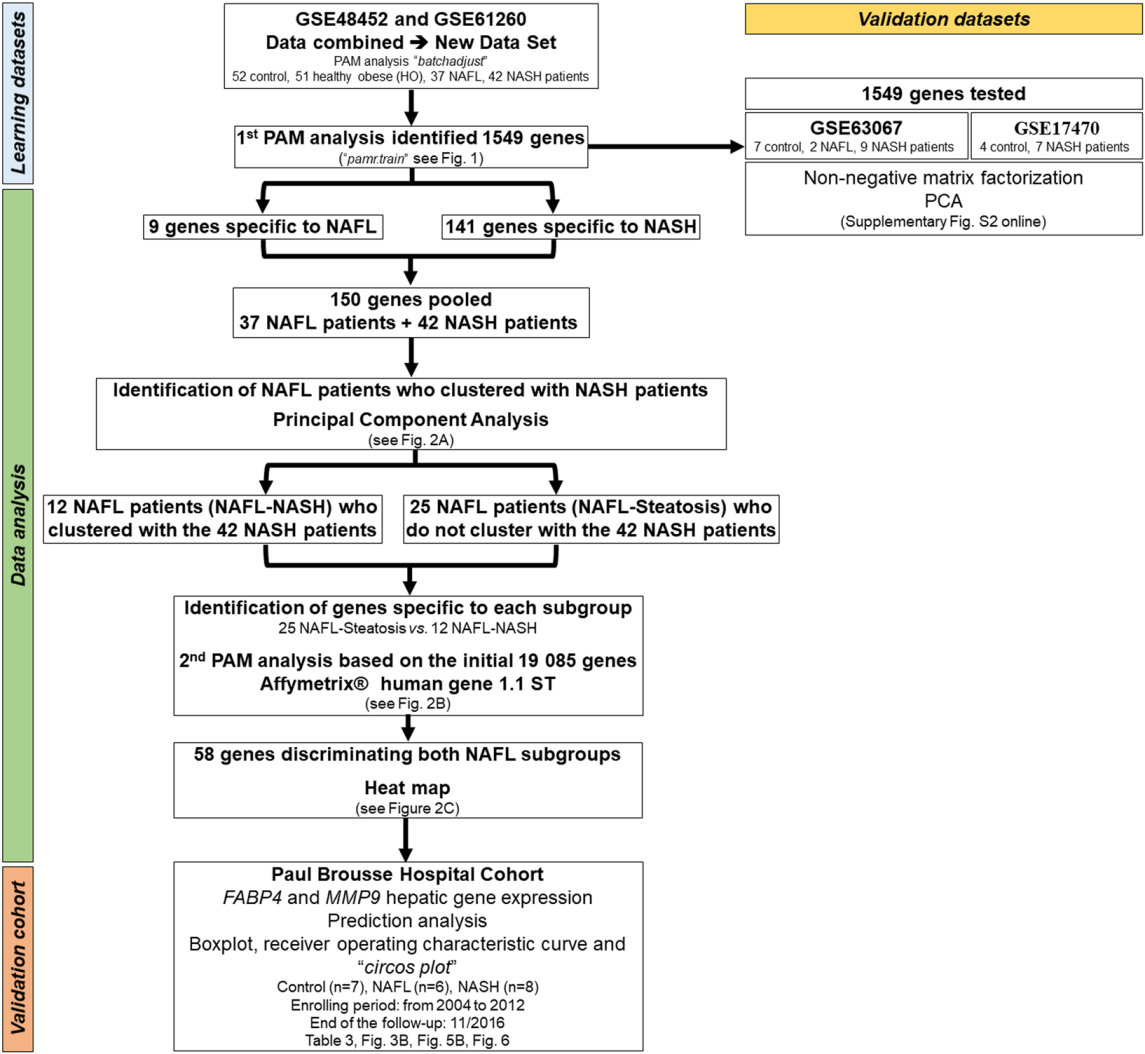
Study flowchart for the identification of 58 genes allowing predicting the subgroup of NAFL patients likely to progress to NASH. The first prediction analysis of microarrays (PAM) performed on the learning datasets (GSE48452 and GSE61260, blue) identified 1,549 genes allowing differentiating the four groups of patients (Controls, heathy obese patients, obese patients with NAFL and obese patients with NASH). The 1,549 genes were then validated using validation datasets (GSE63067 and GSE17470, yellow). Data were then analyzed (green). Nine genes were only specific to obese patients with NAFL and 141 genes were only specific to obese patients with NASH. These 150 genes were pooled, and principal component analyzes were performed in the groups of NAHL and NASH patients. Twelve obese patients with NAFL were misclassified as obese patients with NASH (referred to as NAFL-NASH patients) whereas 25 obese patients with NAFL were not (referred to as NAFL-Steatosis patients). Then, a second PAM of all the 19,085 genes of the microarray chip was performed in the 25 NAFL-Steatosis and 12 NAFL-NASH patients. Fifty-eight genes (54 were upregulated and 4 were downregulated) allowing distinguishing the 12 NAFL-NASH patients and the 25 NAFL-Steatosis patients were identified, as confirmed in the heatmap. Finally, the results were confirmed in the cohort of patients (7 control subjects, 6 NAFL patients and 8 NASH patients) from Paul Brousse Hospital (Validation cohort, orange). The flowchart results are shown in Fig. 2 to Fig. 6 and in Supplementary Fig. S1 to Supplementary Fig. S5.

Data were analyzed using R 3.5.2 software^35^ following the study flowchart summarized in Fig. 1. First, each matrix of data was normalized with robust multi-array average (RMA). Briefly, RMA is an algorithm used to create an expression matrix from Affymetrix® data. The raw intensity values are corrected for background, log2 transformed and then quantile normalized. Then, a linear model is fit to the normalized data to obtain a measure of the gene expression for each probe pair on each array and then combined with structured query language (SQL) request^36^. Then, both batches were normalized using prediction analysis for microarrays (PAM) with “*batchadjust*” implemented in “*PAMR*” for R package at the “*pamr.train*” function^37,38^. Then the threshold was calculated to determine the minimum number of genes allowing distinguishing the four sample groups based on the calculation of the minimum misclassification error for each group leading to a table of true *versus* predicted values (*pamr.confusion*), from a nearest shrunken centroid fit (*pamr.adaptthresh*)^38^. Thereafter, the genes of interest were sorted based on their best score (*pamr.listgenes*) and the gene(s) with the best score which survived the thresholding from the nearest shrunken centroid classifier were plotted (*pamr.geneplot*).

The gene lists generated by the different PAM were compared using the “*Venn*” function in “*gplots*” for R package, indicating the number of overlapping transcripts in each sample group^39^.

### Validation datasets

Two independent human liver biopsy datasets were selected and downloaded (GSE63067 and GSE17470; http://www.ncbi.nlm.nih.gov/geo/) to validate the genes of interest identified using the learning datasets (GSE48452 and GSE61260) after the PAM. Here, gene expression levels were measured using HG-U133_Plus_2.0 array (na22 platform, Affymetrix®) and CodeLink Human Whole Genome Bioarray (GE Healthcare/Amersham Biosciences), respectively^40,41^. The GSE63067 dataset included 7 control samples, 2 NAFL samples and 9 NASH samples^41^. The GSE17470 dataset included 4 control samples and 7 NASH samples^40^. The clinical characteristics of the patients who provided these samples were not available. To validate the genes selected in both validation datasets, two independent unsupervised approaches were used, the non-negative matrix factorization (NMF) and the principal component analysis (PCA). The “*NMF*” (R-package) was used here as an unsupervised clustering method for samples using gene expression microarray data from the validation datasets^42^. PCAs were performed using “*FactoMineR*” for R-package. Each sample and the ellipses centered onto the mean representing the 95% confidence interval (CI) were plotted on the PCA. The probabilities associated with the F-test of the variance analysis along the dimension axes (α = 0.05) were calculated^43^.

### Gene set enrichment analysis

The lists of genes identified at each step of the analysis of learning datasets were used as data entries for computing enrichment with existing lists created from prior knowledge organized into gene-set libraries. We used Enrichr, a freely available integrative web-based and mobile software application (http://amp.pharm.mssm.edu/Enrichr/) that includes 17 gene-set libraries, an alternative approach to rank enriched terms, and various interactive visualization approaches to display enrichment results^44,45^. Among the 17 libraries, we searched WikiPathways 2016 and/or KEGG 2016 (Kyoto Encyclopedia Gene and Genome) libraries. The algorithm calculated the p-value, Z-score and combined score for each signaling pathway identified. Using methods based on the Z-score and combined score has been shown to be the best approach to recover a higher number of correct terms^45^. Thus, only the signaling pathways with a significant p-value (*p-value* <0.05) were ranked based on their combined score and represented as a bar graph and network. Also, to pin-point certain genes associated with a signaling pathway, data were represented as clustergrams for better clarity.

To identify specific molecular signatures, we also used MSigDB database software v5.2 developed by the Broad Institute (http://software.broadinstitute.org/gsea/msigdb/)^46^. KEGG and Reactome databases were used to identify the genes and signaling pathways significantly related to the NASH group from the learning datasets. Data were represented as enriched set plots.

### Unsupervised cluster analyzes

During the identification process of genes specific to a subgroup of samples, to confirm the specific gene expressions in each group, unsupervised cluster analyzes were performed using dendrograms generated from the “*heatmap.2*” function in “*gplots*” for R package. The Z-scores for each gene and sample were calculated and unsupervised cluster analyzes were represented as heat maps.

### Validation cohort

A total of 21 patients who consulted at Paul Brousse hospital between 2004 and 2012 and with a follow-up of at least 4 years were included in this study. They were divided as follows: Controls (n = 7), NAFL patients (n = 6) and NASH patients (n = 8). A histological distinction based on the NAS was used to differentiate NAFL from NASH as previously described^20,21^. Patient clinical and biological characteristics, including the general health status, metabolic syndrome and liver function were retrospectively recorded. Exclusion criteria were patients with liver diseases such as viral hepatitis B, viral hepatitis C, primary biliary cirrhosis, sclerosing cholangitis, autoimmune hepatitis, hemochromatosis, Wilson’s disease, α1-antitrypsin deficiency, drug-induced liver disease and patients with alcohol consumption greater than 20 g/day for women and 30 g/day for men. Our institutional review board (Paul Brousse hospital-Centre des Ressources Biologiques Paris-SUD, CRB Paris Sud, Bio Banking Number: 0033-00089) approved the study conduct and a written informed consent was obtained from all patients. The study was conducted in accordance with the relevant “Declaration of Helsinki” and “International Conference on Harmonization Good Clinical Practice” guidelines and the French ethical laws.

### Quantitative reverse transcription PCR

Total RNA were extracted from frozen liver biopsies using RNA-STAT 60 reagent (AMS Biotechnology Europe LTD). RNA levels and quality were assessed using NanoDrop^®^-ND1000 (Thermo Scientific). cDNAs were generated using RivertAid^®^ First Strand cDNA Synthesis (Thermo Scientific), and Syber Green from FastStart Essential DNA Green Master mixes (Roche, Life Science) were used to quantify hepatic *fatty acid-binding protein-4* (*FABP4*) and *matrix metallopeptidase-9* (*MMP9*) mRNA levels using the gene-specific primers described in Supplementary Table S1.

Q-RT-PCR was performed using the LightCycler^®^ 96 Instrument (Roche, Life Science). Gene expression levels were normalized to *glyceraldehyde-3-phosphate dehydrogenase* (*GAPDH*) mRNA levels and data were analyzed using LightCycler® 96 SW 1.1 software (Roche, Life Science). For each sample, the gene of interest level to *GAPDH* level ratio was calculated based on an arbitrary number of copies determined using the standard curve for each gene, as previously described^47^.

### Q-RT-PCR data analyzes

*FABP4* and *MMP9* hepatic mRNA levels were assessed using a PCA by plotting each patient and the ellipses centered onto the mean representing the 95% CI. The probabilities associated with the F-test of the analysis of variance along the dimension axes (α = 0.05) were calculated. To determine the individual and combined sensitivity and specificity of both markers, receiver operating characteristic (ROC) curves, the area under the curve (AUC), negative and positive predictive values (PV) and optimal response cut-off points (Ir.eta) were assessed using “*pROC*” and “*Epi*” for R package. A one-way ANOVA was used to analyze the distribution between the patient groups using “*beeswarm*” for R package and was represented by boxplots. Patient clinical data were analyzed to assess the predictive *FABP4* and *MMP9* mRNA expression levels. NAFL and NASH patients were divided into two subgroups: patients with low *FABP4* and *MMP9* mRNA levels (NAFL_FABP4_MMP9_L and NASH_FABP4_MMP9_L) and patients with high *FABP4* and/or *MMP9* mRNA levels (NAFL_FABP4_MMP9_H and NASH_FABP4_MMP9_H). The clinical events, defined as a worsening of the liver disease, were recorded during the follow-up for each patient. In the Data were analyzed using a Fisher’s exact test and represented as circular layouts (“*circos plot*”) using “*circlize*” for R package.

### Ethics approval and consent to participate

The institutional review board of the hospital (Paul Brousse hospital-Centre des Resources Biologiques Paris-SUD, Bio Banking Number: 0033-00089) approved the study and written informed consent was obtained from all patients. Access to this material and all experiments were performed in accordance with the relevant guidelines and regulation of the French ethical laws.

## Results

### Clinical characteristics of samples from the learning dataset matrix

For further biostatistical analyzes (Fig. 1), we combined both GSEA (GSE48452 and GSE61260) that have been published previously^33,34^ to create a new learning dataset matrix. The clinical data from this learning dataset were based on the clinical data available in the GEO dataset website and included 52 control subjects, 51 HO patients, 37 obese patients with NAFL and 42 obese patients with NASH classified according to their NAFLD-activity score as previously described^20,21^. The clinical characteristics of the different groups of patients are summarized in Table 1. Briefly, control subjects were older than patients who underwent bariatric surgery in the other groups. Women were significantly more represented in the four groups (*p-value* <0.0001, chi-squared test). Importantly, the number of women was higher in control subjects and HO patients than in obese patients with NAFL and NASH (75/28 *versus* 47/32, *p-value* = 0.0581, chi-squared test), but there was no significant difference in gender distribution between obese patients with NAFL and NASH (21/16 *versus* 26/16; *p-value* = 0.641, chi-squared test). As expected, the BMI was significantly higher in the three groups of obese patients compared to control subjects, with significantly higher serum leptin levels and lower serum adiponectin levels. However, no difference in age, BMI, serum leptin and adiponectin levels was observed between the three groups of obese patients.

To confirm the statistical results from the new learning dataset matrix, we used validation datasets (GSE63067 and GSE17470). The clinical characteristics of the patients included in these validation datasets were not available.

#### Identification of gene signatures and associated signaling pathways according to patient group in the learning and validation datasets

The new learning data matrix combining the four groups of human liver samples from the learning datasets (GSE48452 and GSE61260) was built based on the data from human liver biopsies using the same high-density oligonucleotide microarray version (see Methods)^33,34^. The PCA of the new matrix showed a high variance between the two microarray results of both learning datasets (Supplementary Fig. S1A). Then, the next step was to normalize both datasets to create the learning data matrix. A PAM was then applied to the new matrix using all control subjects from both datasets as “controls” to normalize the variables of the datasets (Fig. 1). We confirmed the homogeneity of the distribution of the patients from both datasets using all genes from the normalized new matrix by performing a PCA (Supplementary Fig. S1B).

**Figure 2:**
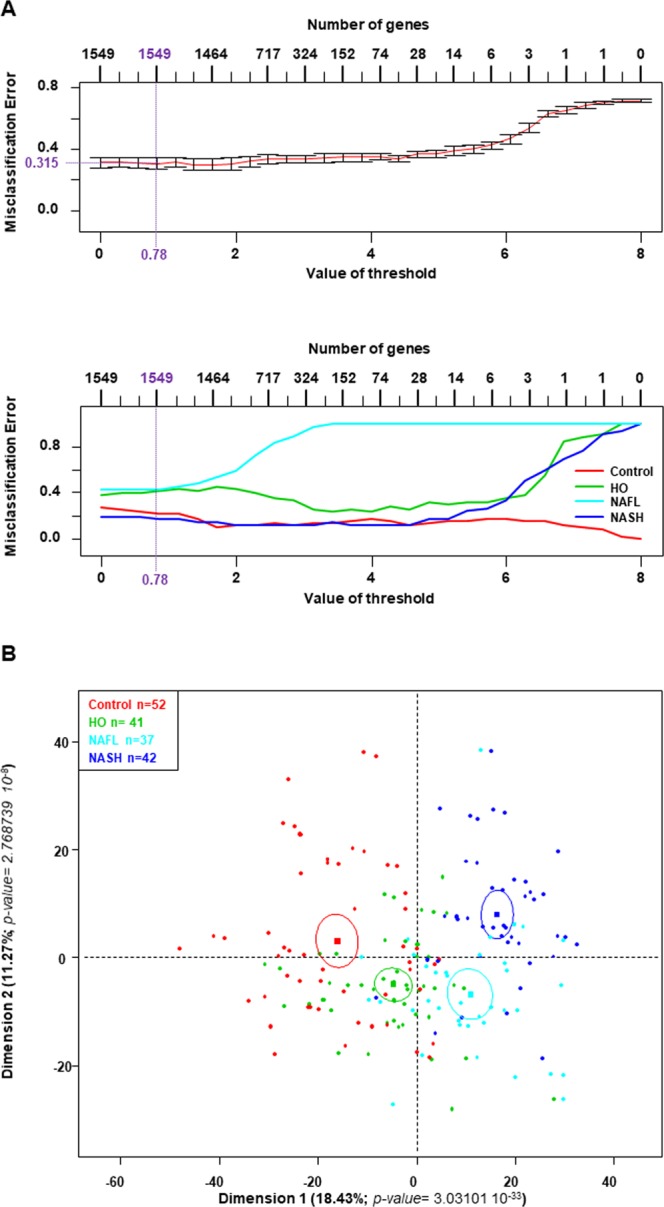
Identification of 1,549 genes differentiating the four groups of patients from the learning datasets (GSE48452 and GSE61260). **(A)** The transcriptome of the four groups of patients from GSE48452 and GSE61260 datasets was analyzed using a prediction analysis of microarrays (PAM) algorithm and 1,549 genes were identified with a threshold of 0.78, corresponding to an overall misclassification error rate of 0.315 (upper panel) and the misclassification error rate for each group of patients is detailed (lower panel). **(B)** Principal component analysis of the four groups of patients differentiated using the 1,549 genes identified. The dots represent each patients from each GSE, the lines are the ellipses centered onto the mean (colored squares) representing the 95% confidence interval, and the *p-value* is the probability associated with the F-test of the analysis of variance along the axes of the first and second dimensions (α = 0.05).

Then, a PAM was performed on the standardized and corrected transcriptome arrays including the 182 samples divided into 4 groups. The PAM identified 1,549 genes with a threshold of 0.78, corresponding to an overall low misclassification error rate of 31.5% (Fig. 2A, upper panel). This gene selection allowed separating most samples in each group, as shown by the cross-validated misclassification error curves (Fig. 2A, lower panel). The confusion matrix showed that obese patients with NAFL (n = 37) had a misclassification error rate of 0.5946 (22/37) with three patients classified as “Controls”, nine patients classified as HO patients and, interestingly, 10 patients classified as NASH patients (Table 2). These observations suggested that at least 10 obese patients with NAFL could be misclassified and could belong to the group of obese patients with NASH.

**Table 2:** Detailed misclassification errors (confusion matrix) in each group of patients from the learning dataset (GSE48452 and GSE61260) according to the prediction analysis of microarrays (Fig. 2A) performed to analyze the new data frame.

	Control	HO	NAFL	NASH	Class Error Rate
Control	**46**	1	3	2	0.1153
HO	13	**29**	6	3	0.4313
NAFL	3	9	**15**	***10***	0.5946
NASH	1	2	2	**37**	0.4313

HO: healthy obese; GSE48452 and GSE61260^33,34^.

The PCA based on the 1,549 genes (Fig. 2B) showed a progression between the four groups of patients based on their gene signature as shown by the first and second dimensions of the PCA, with control subjects on the left panel of the graph followed by HO patients, NAFL patients and NASH patients on the top panel of the graph. These results suggested that among the 1,549 genes identified, some genes could be only specific to a group and not to the other three groups, which could explain the good separation observed in the PCA (Figs. 1, 2B).

Finally, to confirm the signature composed of the 1,549 genes identified, two independent validation datasets (GSE63067 and GSE17470) were used (Fig. 1). A non-negative matrix factorization and a PCA showed that the 1,549 genes also allowed separating each group of patients included in the validation datasets (Supplementary Fig. S2).

Thus, using a batch-adjust normalization approach associated with a gene prediction algorithm to combine two different Affymetrix® microarray datasets, 1,549 genes were selected to distinguish healthy subjects, HO patients, obese patients with NAFL and obese patients with NASH. This gene signature was also confirmed in two other independent GEO datasets. Interestingly, this specific set of genes allowed identifying a subgroup of 10 patients with NAFL among the 37 patients (27.03%) who were classified as NASH patients. These results strongly suggested that these 10 patients with NAFL shared the same specific gene profile as NASH patients and that this specific gene profile could help to identify the process involved in the progression from steatosis to NASH.

#### Identification of gene expression transitions associated with the inflammatory process, the apoptosis pathway and extracellular matrix remodeling

The PAM (Fig. 2A) identified 186 genes that were only specific to control subjects, 12 genes that were only specific to HO patients, 9 genes that were only specific to obese patients with NAFL and 141 genes that were only specific to obese patients with NASH (Supplementary Dataset 1). Then, “Enrichr” online software was used to identify the pathways associated with the genes identified (Supplementary Fig. 3). The 186 control-specific genes were involved in common signaling pathways such as translation driven by ribosomes, and functions such as estrogen signaling and circadian rhythm (Supplementary Fig. S3A). Interestingly, HO patients also expressed genes (n = 12) involved in estrogen signaling, circadian rhythm (*i.e*. serotonin receptors), nuclear receptors and transcription factors, inflammatory process, and more importantly genes involved in the extracellular matrix remodeling (ECMR) (Supplementary Fig. S3B). In both control subjects and HO patients, the identification of genes involved in the estrogen signaling pathway was in line with the over-represented female gender compared to NAFL and NASH patients (75 to 28 *vs*. 47 to 32, *p-value* = 0.0581, respectively Table 1). Importantly, these results validated the biostatistical workflow chosen and showed the sensitivity of combining such biostatistical approaches and the microarray gene expression technology in a large cohort of patients.

The 9 genes specific to obese patients with NAFL were involved in the JAK/STAT, MAPK and PI3K/AKT/mTOR signaling pathways associated with ECMR pathways (Supplementary Fig. S3C). Finally, the 141 genes specific to obese patients with NASH were also involved in ECMR, apoptosis and carcinogenesis pathways (Supplementary Fig. S3D).

Thus, four sets of genes (186, 21, 9 and 141 genes) that were specific to each group of patients were identified. The sets of genes specific to NAFL and NASH patients (9 and 141 genes, respectively) were involved in ECMR, apoptosis and carcinogenesis pathways.

In addition, the signaling pathways in which the genes identified were involved could be found in each group of patients with an increased expression according to disease the severity, from the inflammatory process to ECMR and oncogenic pathways. Thus, these results also validated the biostatistical workflow approaches used to identify these genes.

#### Identification of a subgroup of obese patients with NAFL with a gene signature similar to that of obese patients with NASH according to FABP4 expression levels

As shown, the confusion matrix identified 12 obese patients with NAFL classified as control subjects (n = 3) or HO patients (n = 9), and 10 obese patients with NAFL classified as patients with NASH (Table 2). Also, the gene enrichment pathway analysis showed that some pathways were common to the different NAFLD subtypes (Supplementary Fig. S3). These results suggested that a subgroup of NAFL patients (*i.e*. the 10 patients who were misclassified) had already a gene signature similar to that of NASH patients.

**Figure 3:**
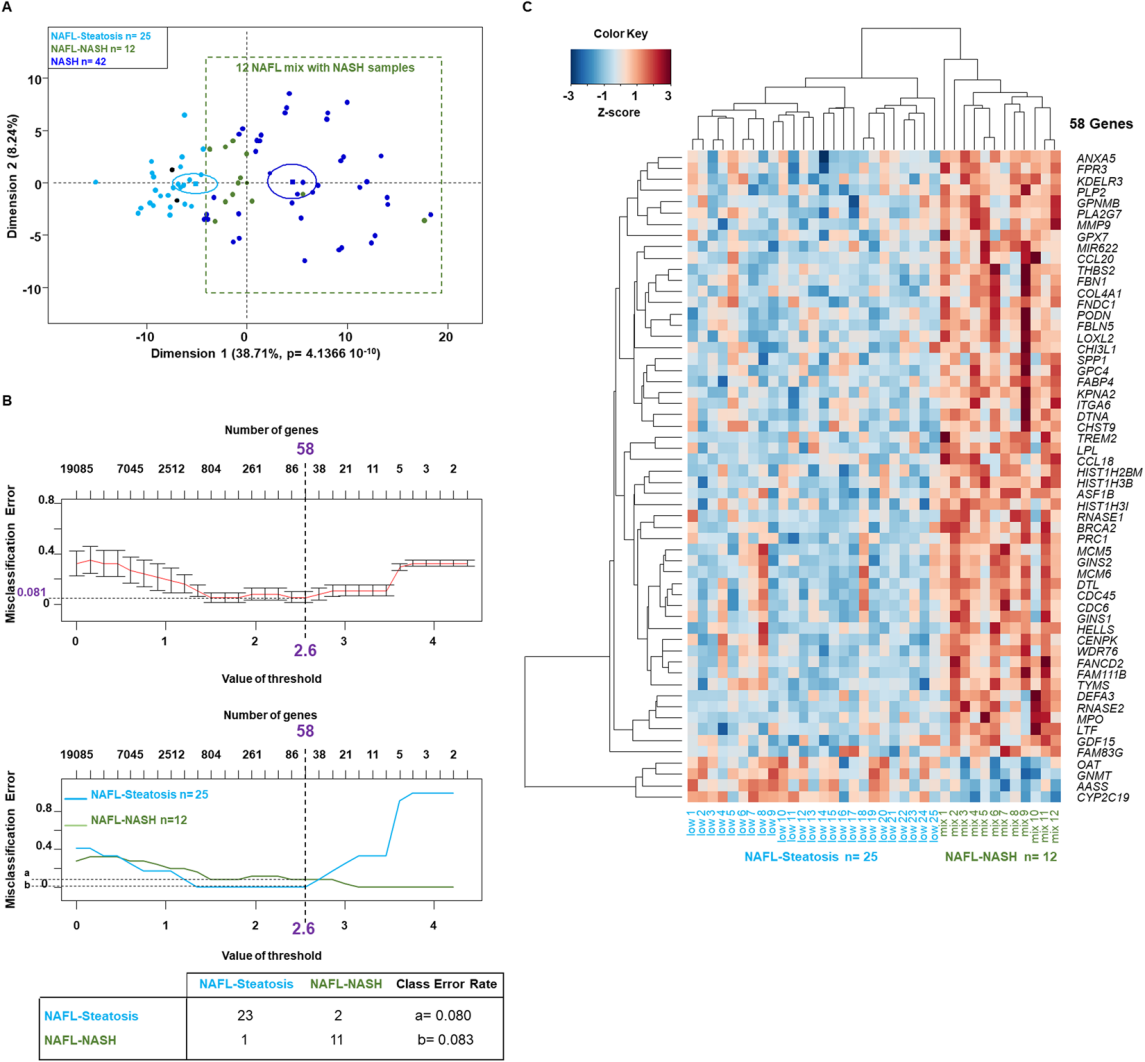
Identification of 58 genes allowing differentiating a subgroup of obese patients with steatosis misclassified as NASH patients. **(A)** Unsupervised analysis of obese patients with NAFL and NASH using a total of 150 genes: 9 genes were specific to NAFL patients and 141 genes were specific to NASH patients compared to the other groups of patients from GSE48452 and GSE61260 datasets. The principal component analysis identified 12 NAFL patients who were misclassified as NASH patients (referred to as NAFL-NASH patients; green dots; dark blue dots) and 25 NAFL patients who were not misclassified as NASH patients (referred to as NAFL-Steatosis patients; light blue dots). The dots represent each patient, the lines are the ellipses centered onto the mean (colored squares) representing the 95% confidence interval, and the p-value is the probability associated with the F-test of the analysis of variance along each axis. Obese patients with NAFL, n = 37; obese patients with NASH, n = 41 according to the original publications. **(B)** The prediction analysis of microarrays identified 58 genes based on the threshold of 2.6 calculated to achieve an optimal misclassification error rate for all groups of 0.081 (upper panel) and for each group of 0.0800 and 0.083 (middle panel, dash lines). The table details the misclassified patients in each subgroup of patients analyzed. **(C)** Heatmap of obese patients with NAFL misclassified in two subgroups of patients based on the 58 genes identified in (**B**) and listed on the right.

To test this assumption, the genes specific to patients with steatosis (n = 9) and NASH (n = 141) were combined to create a new matrix with 150 genes and two groups of patients from the learning datasets (37 + 42 = 79 patients in total, Fig. 1). An unsupervised cluster analysis of this new matrix was then performed and showed that among the 37 obese patients with NAFL, 12 patients were misclassified as obese patients with NASH (Figs. 3A, S4). Then, obese patients with NAFL were split into two groups: patients who were misclassified as obese patients with NASH (NAFL mixed with NASH or NAFL-NASH patients, n = 12) and the remaining NAFL patients (NAFL-Steatosis patients, n = 25). To define a specific gene signature in order to distinguish these two subgroups and to avoid any bias, a PAM was performed using all the genes of the microarrays (n = 19,085). The results showed that the best threshold was 2.6 to achieve a minimum of overall misclassification error rate (0.081) and this threshold allowed identifying 58 genes (Fig. 3B; Supplementary Dataset 2), associated with good results for the misclassification rate as shown by the confusion matrix (Fig. 3B). The unsupervised cluster analysis of the 58 selected genes showed that NAFL livers with a NASH signature (NAFL-NASH) and NAFL (NAFL-Steatosis) patients were clearly distinguishable (Fig. 3C). Among these 58 genes, 54 were upregulated and 4 were downregulated (*OAT*, *GNMT*, *AASS* and *CYP2C19*) in the NAFL-NASH subgroup **(**Fig. 3C; Supplementary Dataset 2). The 4 genes down-regulated in the NAFL-NASH subgroup were mainly involved in metabolic pathways such as amino acid metabolism which can lead to the synthesis of pyruvate and subsequently acetyl CoA, the precursors of linoleic acid and arachidonic acid synthesis (Supplementary Fig. S5A). This finding was in agreement with what is expected in the fatty liver tissue, where the lipid metabolism is dysregulated^48,49^. The 54 genes distinguishing obese patients with steatosis who were misclassified as NASH patients (NAFL-NASH subgroup) were involved in ECMR, DNA regulation (DNA repair, replication, G1/S cell cycle), inflammatory processes and some were involved in insulin resistance and lipid metabolism (Supplementary Fig. S5B).

**Figure 4:**
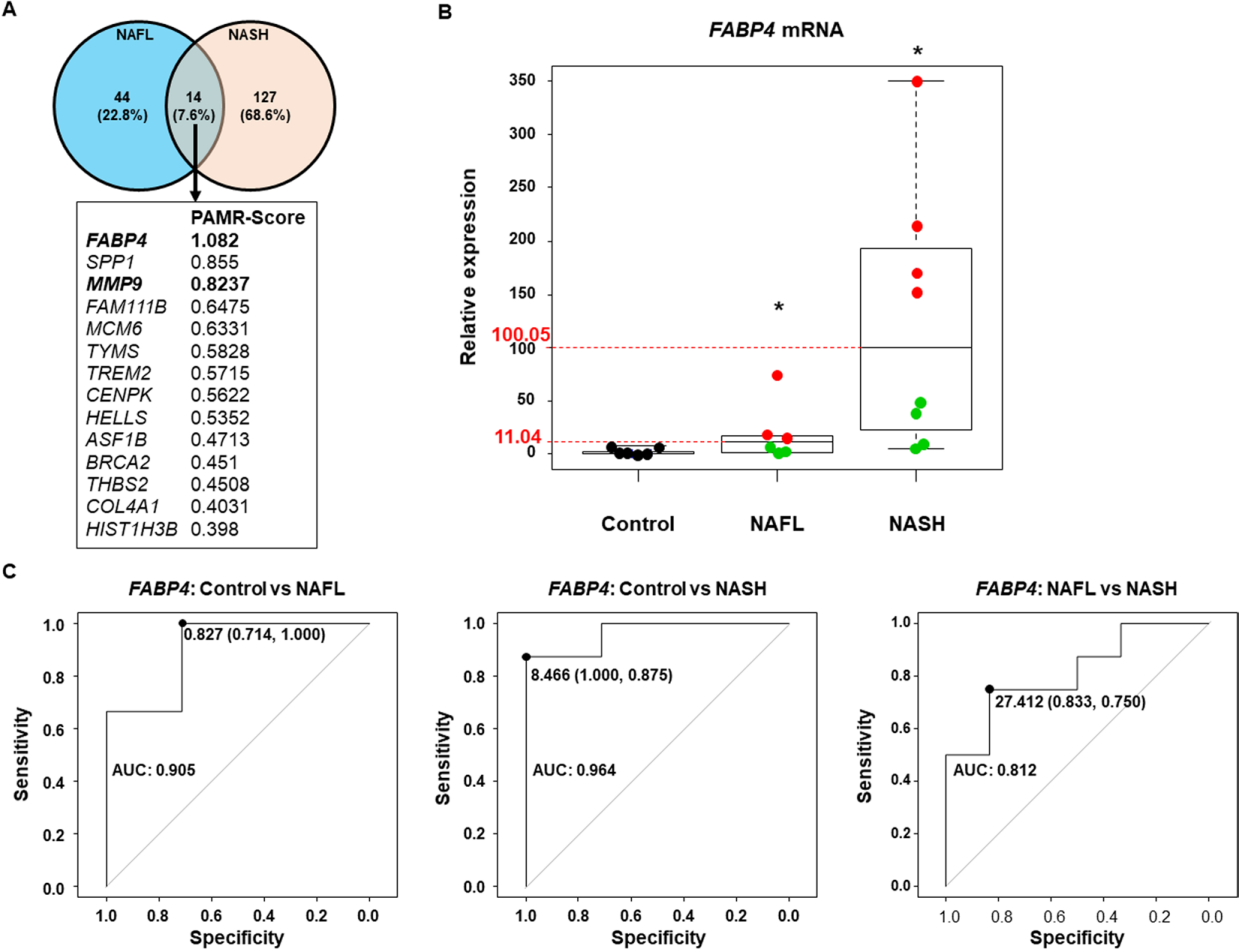
*FABP4* hepatic mRNA expression level distinguished control subjects from patients with NAFL or NASH as well as two subgroups in the NAFL and NASH groups. (**A)** The Venn diagram identified 14 genes among the 58 genes differentiating the two subgroups of NAFL patients (NAFL-Steatosis and NAFL-NASH patients, see Fig. 3C) and among the 141 genes specific to NASH patients. **(B)** Boxplots of *FABP4* hepatic mRNA expression level in Control (n = 7), NAFL (n = 6) and NASH (n = 8) patients from Paul Brousse Hospital (Validation cohort). Identification of two subgroups in NAFL patients and two subgroups in NASH patients based on their median values (11.04 and 100.05, respectively) and represented by green and red dots. *p <0.05 compared to control patients based on an ANOVA. (**C**) Receiver operating characteristic (ROC) curve using *FABP4* hepatic mRNA expression level comparing control versus NAFL patients (left panel), control versus NASH patients (middle panel) and NAFL versus NASH patients (right panel). The values of the Area Under the Curve (AUC) are shown, as well as the threshold, sensitivity, and specificity for the optimal response cut-off points (black dot).

Venn diagrams were used to compare the 58 genes that allowed distinguishing the NAFL-NASH and NAFL-steatosis subgroups to the 141 genes specific to obese patients with NASH. Only 14 genes were common to both gene sets (Fig. 4A). The enrichment analysis of these 14 genes showed that they were involved in ECMR, inflammation and oncogenic pathways as expected (Supplementary Fig. S6A,B). Among the 141 genes identified in obese patients with NASH, the top-ranked gene was *FABP4* (+1.082-fold in the NASH group, Supplementary Dataset 1**)**. *FABP4* was also the top-ranked gene among the 58 genes that allowed distinguishing NAFL-NASH patients from NAFL-Steatosis patients according to the PAM results (+1.79-fold, Supplementary Dataset 2, Fig. 2C). Furthermore, *FABP4* was strongly associated with 4 genes involved in ECMR and oncogenic pathways (*BRCA2*, *COL4A1*, *ITGA6* and *MMP9*, Fig. 4A).

NAFL patients with high *FABP4* mRNA expression level (12 out of the 37 obese patients with NAFL) also expressed high mRNA expression levels for genes involved in ECMR and inflammatory pathways (Fig. 3C). Then, to confirm these results, we used a Validation cohort consisting in 21 patients who consulted in our institution and their clinical characteristics were summarized in Table 3. *FABP4* hepatic mRNA expression level was quantified by Q-RT-PCR and allowed distinguishing the three groups of patients (Fig. 4B). *FABP4* mRNA expression level allowed identifying two subgroups both in NAFL and NASH patients based on their median relative gene expression levels (11.04 and 100.05, respectively; Fig. 4B). *FABP4* mRNA expression levels distinguished two subgroups of patients diagnosed by the pathologist as NAFL or NASH patients (Fig. 4B), but the ROC curves based on *FABP4* expression levels also allowed distinguishing Control subjects from NAFL patients (specificity 0.714; sensitivity 1.000; AUC = 0.905) as well as Control subjects from NASH patients (specificity 1.000; sensitivity 0.875; AUC = 0.964) while no significant difference was observed between NAFL and NASH patients (specificity 0.853; sensitivity 0.750; AUC = 0.812, Fig. 4C). The last result could be due to the high variability in *FABP4* mRNA expression in the NAFL and NASH groups (Fig. 4B). Interestingly, these results strongly suggested that *FABP4* mRNA expression level could help to identify NAFL patients likely to belong to the group of NASH patients and also to identify a subgroup of NASH patients likely to progress to cirrhosis and/or HCC.

**Table 3:** Characteristics of patients from Paul Brousse Hospital (*i.e*. Validation cohort)

	Control n = 7	NAFL n = 6		NASH n = 8		NAFL n = 6		NASH n = 8	
	Low *FABP4* and *MMP9* levels	Low *FABP4* level	High *FABP4* level	Low *FABP4* level	High *FABP4* level	Low *MMP9* level	High *MMP9* level	Low *MMP9* level	High *MMP9* level
Gender F/M	5/2	1/2	1/2	2/2	4/0	0/3	2/1	3/1	3/1
Age (year)	32.4 ± 6.6	54.3 ± 9.4	56.0 ± 8.9	51.3 ± 5.5	60.8 ± 5.9	45.3 ± 7.4	65 ± 4.2	55.7 ± 6.8	57.5 ± 6.1
BMI (kg/m^2^)	24.5 ± 0.9	23.1 ± 0.6	27.6 ± 3.1	35.5 ± 2.4	32.9 ± 3.2	24.4 ± 3.1	32.5 ± 2.0	33.5 ± 2.4	34.5 ± 3.3
Glycemia (mol/L)	5.3 ± 0.2	8.1 ± 1.9	6.5 ± 1.2	6.4 ± 0.4	7.2 ± 1.9	5.4 ± 0.6	9.7 ± 0.2	5.5 ± 2.0	8.3 ± 0.4
ALT (IU/L	20.3 ± 2.5	41.5 ± 5.3	32.0 ± 5.2	46.7 ± 5.5	50.8 ± 7.9	30.0 ± 2.3	44.5 ± 3.6	50.8 ± 6.9	46.8 ± 7.2
AST (IU/L)	24 ± 2.2	28.0 ± 6.5	35.3 ± 9.5	40.0 ± 8.0	53.3 ± 3.3	24.0 ± 7.3	45.0 ± 2.6	48.5 ± 2.8	46.3 ± 8.5
γ-GT (IU/L)	29.3 ± 1.9	94.5 ± 61.6	61.3 ± 11.7	57.3 ± 18.5	219.3 ± 127.0	53.3 ± 51.8	106.5 ± 18.6	81.8 ± 36.5	240.7 ± 150.8
ALP (IU/L)	86.2 ± 7.1	89.0 ± 22.1	92.3 ± 16.7	79.6 ± 8.8	122.8 ± 40.3	92.0 ± 21.6	89.5 ± 17.0	85.5 ± 48.0	129.3 ± 11.6
Creatinine (µmol/L)	71.8 ± 5.9	74.5 ± 9.4	82.7 ±	81.0 ±	63.5 ±	71.3 ± 4.5	91.5 ± 8.3	57.3 ± 15.9	89.3 ± 1.1
**Steatosis**									
S0/S1/S2/S3	7/0/0/0	0/1/2/0	0/1/1/1	0/0/0/4	0/0/1/3	0/0/2/1	0/2/1/0	0/0/0/4	0/0/1/3
NAFLD activity score (NAS)	0.6 ± 0.2	2.3 ± 0.7	3.3 ± 0.7	6.0 ± 0.4	5.8 ± 0.5	2.7 ± 0.9	3.0 ± 0.6	6.0 ± 0.4	5.8 ± 0.5
**Fibrosis**									
0/1a/1b/1c/2/3	5/2/0/0/0/0	0/1/2/0/0/0	0/1/2/0/0/0	0/2/0/2/0/0	0/4/0/0/0	0/0/3/0/0/0	0/2/1/0/0/0	0/3/0/1/0/0	0/3/0/1/0/0
Follow-up duration (years)	7.7 ± 3.2	9.3 ± 1.3	4.3 ± 0.3	5.0 ± 0.6	4.0 ± 0	8 ± 2.3	5.7 ± 1.2	4.0 ± 0	4.5 ± 0.5
Number of clinical events* during the follow-up	0	1	2	1	3	0	3	1	3

Characteristics of patients from Paul Brousse Hospital (*i.e*. Validation cohort) in whom relative *FABP4* and *MMP9* hepatic mRNA expression levels were assessed by Q-RT-PCR. The cohort of patients was based on frozen liver biopsies available at the time of the study. The follow-up duration ranged from 4 to 12 years (samples were collected from 2004 to 2012 and the follow-up was stopped in 2016). The patients were classified based on their NAFLD activity score (NAS) and the subgroups were classified based on their relative *FABP4* and *MMP9* mRNA expression levels. *In NAFL patients, 2 patients developed steatofibrosis, 1 developed NASH, 1 died from a non-liver cause and the other 3 patients did not experience disease progression. In NASH patients, 1 patient developed cirrhosis, one developed HCC with cirrhosis, 1 developed HCC (one nodule) and 1 developed a cholangiocarcinoma and the other patients did not experience disease progression (no change in NAS). Data are presented as a mean ± SEM. The grey boxes represent the biological data increased compared to the groups with Low *FABP4* level or Low *MMP9* level (*p-value* <0.05; unpaired *t*-test). ALT: alanine aminotransferase; AST: aspartate aminotransferase; F: female; γ-GT: gamma-glutamyl transferase; m: male; NAFL, nonalcoholic fatty liver; NASH, nonalcoholic steatohepatitis.

Thus, we characterized a gene signature allowing predicting what patients with steatosis could progress to NASH.

#### Identification of a subgroup of NASH patients with a more aggressive gene profile according to MMP9 mRNA levels

**Figure 5:**
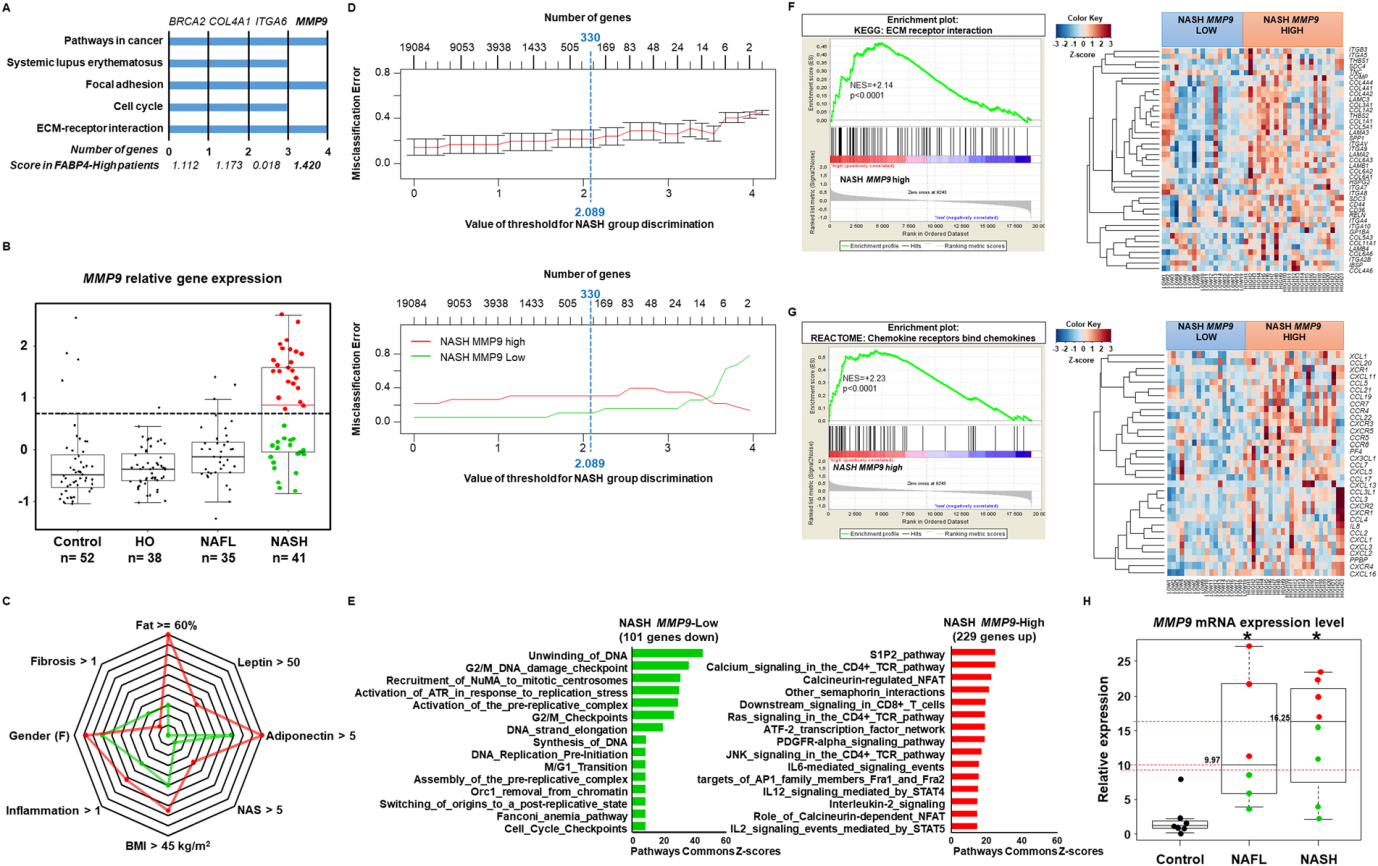
*MMP9* expression level differentiated two subgroups of NASH patients expressing genes predicting a poor disease outcome. (**A)** Gene pathway analysis results based on the 58 genes represented as clustergrams, including the score based on the PAMR analysis for each gene of interest: *BRCA2*, *COL4A1*, *ITGA6* and *MMP9*. **(B)** Relative *MMP9* mRNA expression level in each group of patients from the learning dataset (GSE48452 and GSE61260). Data are represented as boxplots. The threshold represents (dotted line) the 95% confidence interval of the control subjects and identified 23 patients with high relative *MMP9* expression level (red dots) and 19 patients with low relative *MMP*9 expression level (green dots). Control subjects, n = 52; HO: healthy obese patients, n = 38; obese patients with NAFL, n = 35; obese patients with NASH, n = 41. ECMR: extracellular matrix remodeling pathways; hsa: homo sapiens. **(C)** Clinical characteristics of the two subgroups of patients expressing high *MMP9* level (red, n = 22) and low *MMP9* level (green, n = 19). **(D)** The prediction analysis of microarrays identified a minimum set of 330 genes (101 were downregulated and 229 were upregulated) based on the optimal misclassification error rate differentiating the two subgroups of patients expressing respectively high *MMP9* level (red line) and low *MMP9* level (green line). **(E)** The genome set pathway analysis showed that the 229 genes upregulated in the subgroup of patients with high *MMP*9 level were involved in more aggressive pathways compared to the 101 genes downregulated in the other group of NASH patients. The gene set enrichment analysis of the 229 genes upregulated showed genes involved in **(F)** extracellular matrix remodeling (ECMR) and, **(G)** chemokines and chemokine receptors involved in carcinogenesis in NASH patients with high *MMP*9 expression level. Data are presented as enrichment plots using Kyoto encyclopedia of genes and genomes (KEGG) and REACTOME databases in the left panels and as heatmaps with details of genes in the right panels.

From the learning datasets, 13 other genes were associated with high *FABP4* expression levels in NAFL-NASH and NASH patients (Fig. 4A). Among them, four were involved in ECMR: *BRCA2*, *COL4A1*, *ITGA6* and *MMP9*. *MMP9*, a gene encoding for a matrix metalloproteinase, showed the highest scores (fold of +0.8237, Fig. 4A and +1.420, Fig. 5A).

Also, the 14 genes common to NAFL-NASH and NASH patients were involved in PI3K/AKT/mTOR, inflammatory, ECMR and oncogenic pathways (Supplementary Fig. S6) suggesting a progression from NAFL to NASH. Thus, high *MMP9* expression levels could be associated with the progression from steatosis to NASH and possibly with the progression from NASH to HCC or cirrhosis (Figs. 5A and S6). According to epidemiological studies, at least one third of NASH patients would progress to cirrhosis and HCC^1,3–5,41^, that is why we investigated *MPP9* mRNA expression level in the four group of patients from the learning datasets (GSE48452 and GSE61260). As expected, *MMP9* mRNA expression levels allowed distinguishing NASH patients from control subjects, and HO patients and NAFL patients (*p*-value <0.05, ANOVA, Fig. 5B). Furthermore, the boxplot analysis of the 4 groups of patients showed that at least 90% of control subjects, HO patients and NAFL patients expressed low *MMP9* mRNA expression levels, when a threshold corresponding to the third quartile of control subjects was selected. According to this threshold, this analysis showed that *MMP9* mRNA expression levels allowed distinguishing two subgroups of NASH patients, one with high *MMP9* level (23 patients) and another with low *MMP9* level (19 patients, Fig. 5B).

#### Identification of a subgroup of NASH patients with a more aggressive gene profile according to MMP9 mRNA levels

Then, we focused on the two subgroups of NASH patients distinguished by their *MMP9* mRNA expression level (Fig. 5B). The analysis of the clinical and biological outcomes of the subgroups of NASH patients from the learning datasets showed that the subgroup with high *MMP9* mRNA level had higher hepatic fat content, inflammation, BMI, NAS, leptin and adiponectin levels and this subgroup mainly included female patients (Fig. 5C).

Then, the PAM of both subgroups and the whole transcriptome (n = 19,085 genes) identified 330 predictive genes (229 were upregulated and 101 were downregulated) for NASH patients with high *MMP9* level (Fig. 5D, Supplementary Dataset 3). Using GO-Elite software associated with the Pathway Commons database to analyze the matrix of 330 genes, we showed that the 101 genes down-regulated in NASH patients with high *MMP9* level were related to pathways involved in G2/M DNA damage and cell cycle checkpoints, whereas the 229 genes upregulated in these patients were involved in inflammatory processes including the T-cell receptor signaling (TCR) pathway, MAPK, JNK, p38 activation and leading to CD4+ and CD8+ T cell activation and interleukin (IL)2, IL6, and IL12 secretion, the nuclear factor activated T-cells (NAFT) calcium-calcineurin pathway and hypoxia pathways and genes related to the AP1 complex (*e.g*. FRA1 and FRA2 family members). These results showed that, in the one hand, cell signaling pathways, and especially the G2 DNA damage checkpoint, were inhibited, allowing the resumption of cell cycling and entry into mitosis^50^. On the other hand, inflammatory processes were activated with T-cell and interleukin activation (Fig. 5E, Supplementary Dataset 3).

Also, the gene set enrichment analysis using the KEGG database in NASH patients with high *MMP9* expression levels significantly identified ECMR pathways (NES = +2.14, *p-value* <0.0001; Fig. 5F, Supplementary Dataset 4) as shown by the unsupervised cluster analysis and represented by the associated heatmap. These ECMR pathways were associated with a significantly increased expression of genes involved in the CXR/CCL chemokine pathway (Fig. 5G), including *CD44* and *CXCR4* which are cancer stem-cell markers. The expression of these genes is high during the epithelial-mesenchymal transition and they may participate in liver stromal remodeling, like during the progression from cirrhosis to liver cancer, as shown by the enrichment plot analysis and by the associated genes shown in Fig. 5G and confirmed by the unsupervised cluster analysis **(**Supplementary Excel Table S5) and represented by the associated heatmap (Fig. 5G, right panel). These results strongly suggested that NASH patients with high *MMP9* mRNA expression level could also express high levels of genes related to cirrhosis and HCC progression.

To confirm these data, *MMP9* mRNA expression level was quantified in liver biopsies from patients treated at Paul Brousse Hospital and included in the Validation cohort. The data are shown in Fig. 5H. Two subgroups of NAFL patients and two subgroups of NASH patients could be distinguished based on their median *MMP9* mRNA expression levels (9.97-fold and 16.25-fold mRNA expression level, respectively).

#### High FABP4 and MMP9 mRNA expression levels are associated with a poor prognosis in NAFLD or NASH patients

The analysis of genes identified in NAFL-Steatosis patients and NAFL-NASH patients (n = 58 genes, see Fig. 3C) and in NASH patients (n = 141 genes) with high MMP9 level showed that 4 genes, *FABP4*, *MMP9*, *HELLS* and *TREM2*, were shared between these three groups of patients (Fig. 4A). *HELLS* and *TREM2* are involved in global immune responses. More importantly, *FABP4* has been shown to have pleiotropic effects in steatosis, NAFLD, insulin resistance and metabolic syndrome as well as in cell differentiation and chronic inflammation through macrophage activation^51^. Also, *MMP9* is a protein that induces cancer cell invasion and metastasis. Therefore, *MMP9* expression is also considered as a prognostic marker during cancer progression^52,53^. High *MMP9* mRNA expression levels were associated with the expression of genes involved in cancer progression: *BRCA2*, *COL4A1* and *ITGA6*, and were also associated with high *FABP4* mRNA expression levels (Figs. 4A and 5A).

**Figure 6:**
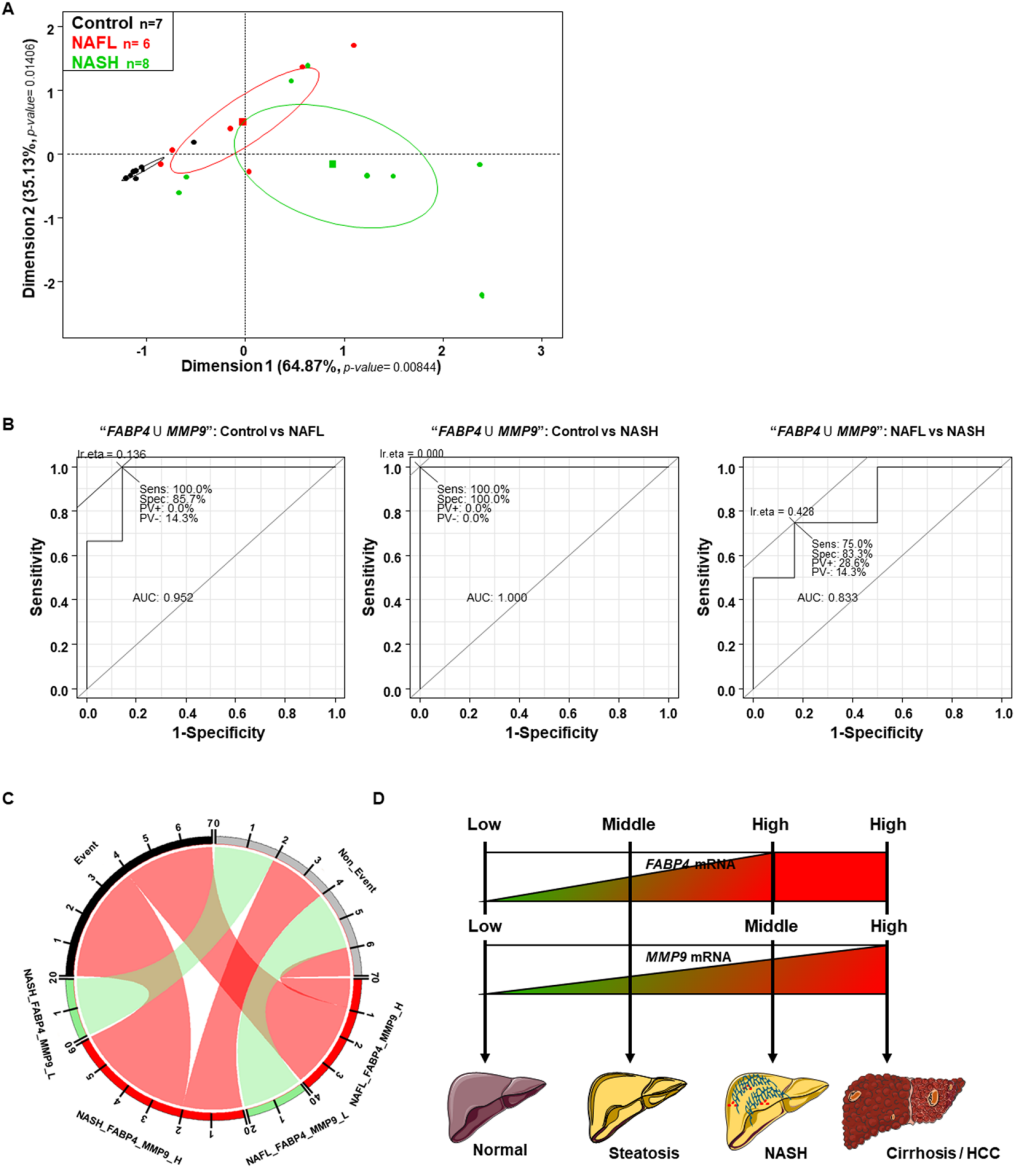
High *FABP4* and *MMP9* hepatic mRNA expression levels predicted poor disease outcomes. (**A)** Principal component analysis of *FABP4* and *MMP9* hepatic mRNA expression levels in three groups of patients from the Validation cohort: 7 control subjects, 6 NAFL patients, and 8 NASH patients. **(B)** Receiver operating characteristic (ROC) curves using *FABP4* and *MMP9* hepatic mRNA expression levels as markers to compare control *versus* NAFL patients (left panel), control *versus* NASH patients (middle panel) and NAFL *versus* NASH patients (right panel). The values of the Area Under the Curve (AUC) are shown, as well as the sensitivity (Sens), specificity (Spec) and predictive values (PV) for the optimal response cut-off points (Ir.eta). **(C)** Circular layout between patients with NAFL or NASH (with at least high *FABP4* and/or *MMP9* mRNA expression levels or with low (L) *FABP4* and *MMP9* mRNA expression levels) and disease worsening (Event) or not (Non-Event). **(D)** Schema showing that patients with steatosis with high *FABP4* hepatic mRNA expression levels were more likely to progress to NASH and that NASH patients with high *MMP9* hepatic mRNA expression levels were more likely to progress to a poorer condition such as cirrhosis and ultimately to hepatocellular carcinoma (HCC).

Then, to determine if *FABP4* and *MMP9* mRNA expression levels could be used as prognostic markers in NAFL and/or NASH patients, we analyzed their expression levels in our Validation cohort (Fig. 1) using a ROC curve analysis. The individual expression levels of *FABP4* and *MMP9* did not allow clearly distinguishing the three groups of patients, in particular NAFL patients from NASH patients (Supplementary Fig. S7). However, when both markers were combined, the unsupervised PCA and ROC curves showed a distinction between Control subjects, NAFL patients and NASH patients (Fig. 6A,B).

The boxplots analyzes of *FABP4* and *MMP9* expression levels from the GSE datasets or the RT-Q-PCR analyzes showed that some NAFL and NASH patients had high or low *FABP4* and *MMP9* mRNA expression levels (Figs. 4B, 5B,H) and a misclassification of patients from the Validation cohort was observed in each group of NAFL and NASH patients as shown by the CART (characteristic and regression tree) analysis (Supplementary Fig. S7C,D, respectively). When *FABP4* and *MMP9* mRNA expression levels were plotted on the same graph with thresholds defined by the median value of each group or by a CART analysis, 4 NAFL patients and 6 NASH patients expressed high *FABP4* and/or *MMP9* mRNA levels (Supplementary Fig. S7E).

Interestingly, a retrospective clinical study of these two subgroups of NAFL and NASH patients showed that 3 NAFL patients had poor outcomes (2 had steatofibrosis, 1 had NASH, 1 died but it was not related to the liver failure) and 4 NASH patients had poor outcomes (1 had cirrhosis, 1 had HCC with cirrhosis, 1 had HCC, 1 had cholangiocarcinoma). The other patients did not experience any progression with no change in NAFL score or NAS. The clinical characteristics of these patients are summarized in Table 3, together with their *FABP4* and *MMP9* hepatic mRNA levels and demonstrated by the *Circos* (Fig. 6C).

Thus, we demonstrated that *FABP4* and *MMP9* hepatic mRNA expression levels could be used as predictive markers for clinical outcomes in patients with NAFL and/or NASH and we were able to build a prediction model (Fig. 6D).

## Discussion

Using publicly available gene expression data from liver biopsies from NAFLD patients, we identified gene markers for the progression from NAFL to NASH and from NASH to cirrhosis/HCC. For a few years, the use of previously published gene expression omnibus datasets has been an approach to identify more reliable diagnostic and/or prognostic markers in cancer^54–56^. Therefore, our approach avoided conducting a new clinical trial, and it allowed significantly increasing the number of patients per group while having well-defined groups of patients. Indeed, our cohort is one of the largest cohorts of well-defined NAFLD patients investigated so far. Furthermore, we confirmed our data in a smaller cohort of patients, which could be considered a more ethical approach.

Using an unbiased machine-learning approach of prediction analysis of microarray data, we combined two independent GEO datasets from two independent cohorts of patients, GSE48452 and GSE61260^33,34^, to create the largest matrix of NAFLD patients associated with gene expression microarray data. Then, a PAMR batch-adjust algorithm was used for both GSE to avoid introducing multiplicative and systematic biases at each step of the microarray experiments and between two or more independent experiments that were performed on the same microarray platform, which led to new biological findings with increased statistical power^57^. This way, we identified 1,549 genes allowing differentiating the four groups of patients and we confirmed these data in two independent human GEO (GSE63067 and GSE17470)^40,41^. Interestingly, the data showed that 27.03% of NAFL patients (10 out of 37 patients) were re-classified in the group of HO patients. As expected, this result confirmed what was already known: the histological scoring system is limited to classify and predict the outcome of NAFL patients. Indeed, we identified 58 genes allowing differentiating two subgroups of patients with NAFL. Fifty-four out of the 58 genes were upregulated in one third of the NAFL patients (12 out of the 37 patients). These genes were involved in inflammatory and ECMR processes as previously reported in NASH patients^32,58–63^. For the first time, we identified 58 genes among which *FABP4* showed the highest expression levels in NAFL patients. *FABP4* was co-expressed with genes involved in NASH progression. Then, these 58 genes expressed in patients with steatosis allowed predicting the outcome of these patients, which could help to improve the follow-up and lead to the implementation of an early therapeutic strategy.

Then, we identified 330 genes specific to a subgroup of NASH patients characterized by high *MMP9* mRNA expression levels. Interestingly, this subgroup of patients had poor outcomes and expressed genes involved in ECMR, inflammation, and carcinogenesis, whereas patients with low *MMP9* mRNA expression levels had gene-expression profiling associated with a better outcome.

Finally, we quantified *FABP4* and *MMP9* hepatic mRNA expression levels in patients who consulted in our institution to confirm if these two genes could be used as prognostic markers. As these prognostic markers were identified in a large cohort of NAFLD patients, this validation cohort was deliberately smaller. Thus, we confirmed retrospectively that *FABP4* and *MMP9* hepatic mRNA expression levels predicted patient clinical outcome. Indeed, elevated *FABP4* hepatic expression levels have been found to positively correlate with NAFLD severity in a human cohort^51,64,65^. Interestingly, high FABP4 serum levels have been reported in NAFLD patients but its use as a prognostic marker in the serum is still controversial^66–70^.

For a decade, MMP9 has been involved in the development and progression of human HCC metastasis^71–74^. More recently, *MMP9* polymorphisms have been associated with the risk of NAFLD and obesity^75^. Therefore, our data showed that *MMP9* hepatic mRNA levels could be used earlier as a prognostic marker to identify NASH patients whose disease could progress to cirrhosis and HCC. To note, a higher MMP-9 serum level has been reported in NAFLD patients compared to control patients but it did not allow distinguishing NAFL patients from NASH patients due to the small sample size and because the authors have not studied patient subgroups^76^. However, D’Amico and colleagues have previously shown significantly higher MMP-9 plasma levels in NASH patients compared to hepatitis C-infected patients with liver disease^77^. In addition, MMP-9 levels have been associated with an increase in inflammatory biomarker levels^78^. Thus, FABP4 and MMP-9 serum levels could be used as non-invasive prognostic markers, especially to identify subgroups of NAFL and NASH patients likely to experience disease progression or not. The next step will be to quantify FABP4 and MMP-9 serum levels in a larger cohort of NAFLD patients to confirm whether these two markers could be used as non-invasive markers.

## Conclusion

In conclusion, using publicly available GEO datasets and an original machine-learning analysis, we identified gene signatures that could help to determine the outcome of patients with steatosis likely to progress to NASH, and the outcome of patients with NASH likely to progress to cirrhosis and/or HCC. Liver biopsies cannot be avoided, however, the use of predictive markers could strongly reduce the number of biopsies during patient follow-up and enable a better management of the patients. Thus, we identified a predictive gene signature in human liver that could be used in patient clinical follow-up, as well as in clinical trials focused on the development of drugs to treat NASH patients, including two main genes, *FABP4* and *MMP9*, the proteins of which could be quantified in the serum and used as non-invasive prognostic markers for NAFL and NASH progression.

## Supplementary information

Supplementary Information

Supplementary Dataset 1

Supplementary Dataset 2

Supplementary Dataset 3

Supplementary Dataset 4

## Data Availability

GSE48452, GSE61260, GSE63067 and GSE17470 have been selected from genome expression omnibus (GEO) database repository on NCBI website (http://www.ncbi.nlm.nih.gov/geo/) and already published^33,34,40,41^. All data generated or analyzed during this study are included in this published article and its Supplementary Information Files including the Excel tables from the biostatistical analysis.
