# Supplementary material for: FABP4 and MMP9 levels identified as predictive factors for poor prognosis in patients with nonalcoholic fatty liver using data mining approaches and gene expression analysis
Source: Sci Rep. 2019 Dec 24;9:19785. doi: 10.1038/s41598-019-56235-y (PMC6930227; doi:10.1038/s41598-019-56235-y)
Supplement: Supplementary file 1 — Supplementary Information [file 41598_2019_56235_MOESM1_ESM.pdf]

FABP4 and *MMP9* levels identified as predictive factors for poor prognosis in patients with nonalcoholic fatty liver using data mining approaches and gene expression analysis

Audrey Coilly[1,2,3,4*], Christophe Desterke[2,3,5*], Catherine Guettier[1,2,3,6], Didier Samuel[1,2,3,4], Franck Chiappini[1,2,3,7]

[1] Inserm, UMR-U1193, Villejuif, F-94800, France

[2] Univ Paris-Sud, Institut André Lwoff, Villejuif, F-94800, France

[3] DHU Hepatinov, Villejuif, F-94800, France

[4] AP-HP, Centre Hépatobiliaire, Hôpital Paul Brousse, Villejuif, F-94800, France

[5] Inserm, UMR-935, Villejuif, F-94800, France

[6] AP-HP, Service d'Anatomopathologie, Hôpital Bicêtre, Le Kremlin-Bicêtre, F-94275, France.

[7] Laboratoire Croissance, Régénération, Réparation et Régénération Tissulaires (CRRET)/ EAC CNRS 7149, Univ Paris-Est Créteil (UPEC), F-94010 Créteil, France

* Both contributed equally to this work.

Supplementary Information

Supplementary Table 1: List of primers used for Q-RT-PCR.

**Genes**	**NCBI Accession number**	**Primer Forward 5'-3'**	**Primer Reverse 5'-3'**	**TM °C**	**Size bp**
*FABP4*_human [1]	NM_001442.2	GCCAGGAATTTGACGAAGTCAC	TTCTGCACATGTACCAGGACAC	59	88
*MMP9*_human	NM_004994.2|	GAGTTCCCGGAGTGAGTTGA	AAAGGTGAGAAGAGAGGGCC	59	225
*GAPDH*_human	NM_002046	CTGACTTCAACAGCGACACC	GTGGTCCAGGGGTCTTACTC	59	172

Primers were design based on the mRNA sequence found in Uniprot database and using Primer3 software and BLASTed (Basic Local Alignment Search Tool) on https://genome.ucsc.edu genomes

FABP4: fatty acid binding protein; *MMP9*: matrix metalloprotease 9; *GAPDH*: glyceraldehyde 3-phosphate dehydrogenase.

1 Elmasri H, Karaaslan C, Teper Y, Ghelfi E, Weng M, Ince TA, *et al.* Fatty acid binding protein 4 is a target of VEGF and a regulator of cell proliferation in endothelial cells. FASEB journal : official publication of the Federation of American Societies for Experimental Biology 2009;**23**:3865-73.

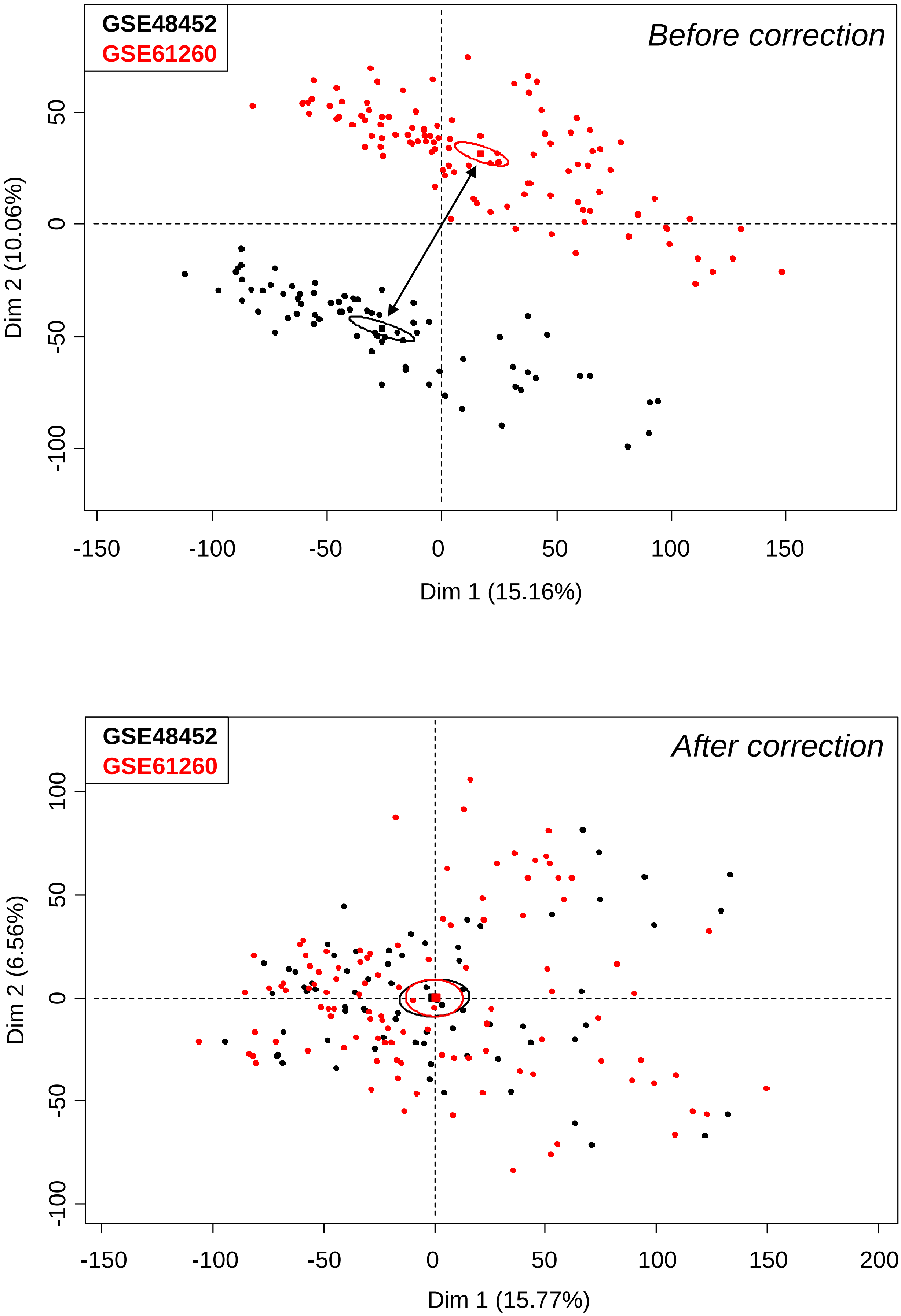

Supplementary Figure S1. Normalization of both GSE48452 and GSE61260 batches. Principal component analyses of GSE48452 and GSE61260 **(A)** before (batch effect p-value= 4.393814 10^{-58}) and **(B)** after batch adjust using prediction analysis of microarray (PAM) algorithm.

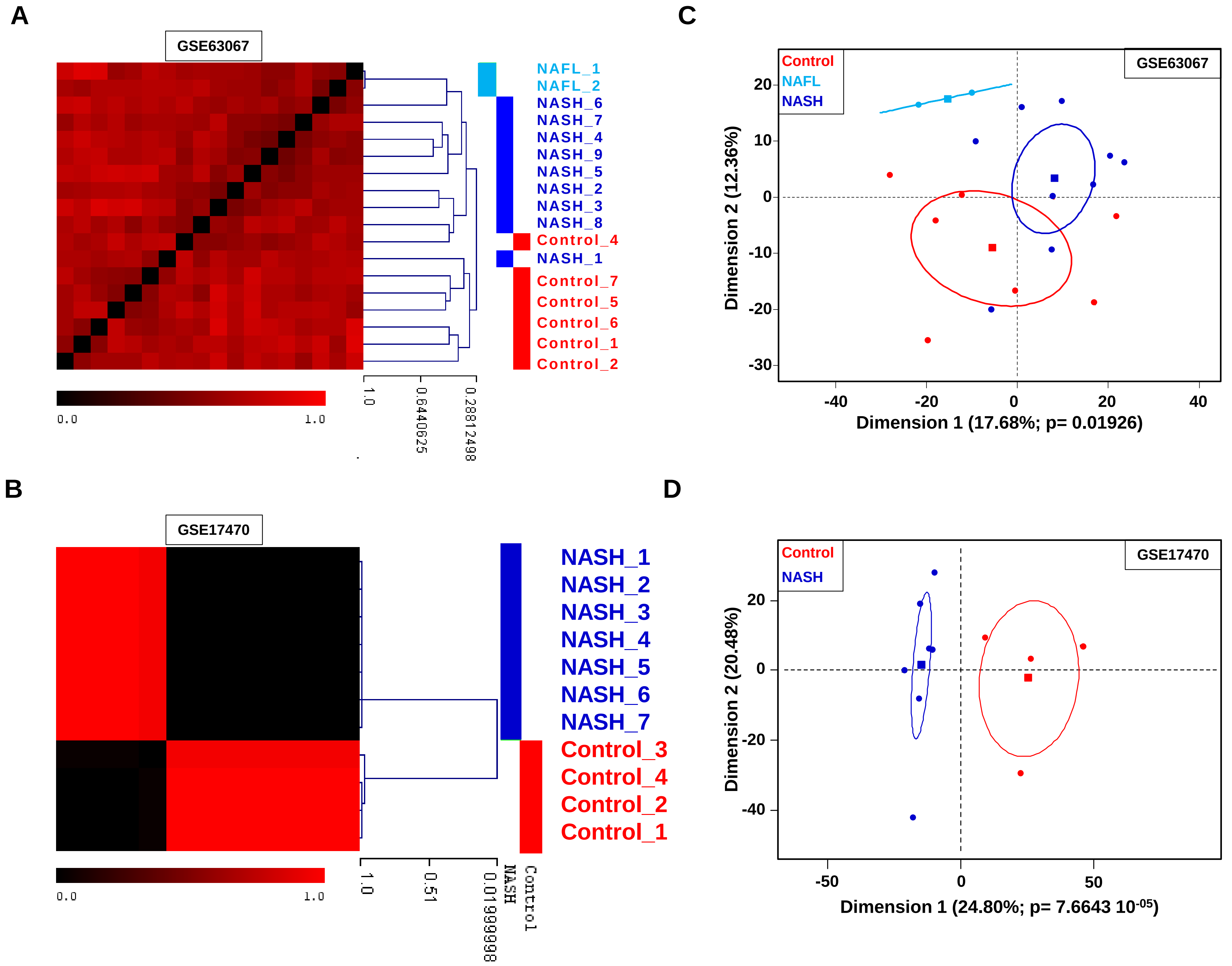

Supplementary Figure S2. The 1549 genes identified have been validated in two other independent gene expression omnibus datasets. (A,B) Non-negative matrix factorization and the corresponding **(C,D)** principal component analyses of both (A,C) GSE63067 and (B,D) GSE17470 using the 1549 genes identified by the learning dataset composed by both GSE48152 and GSE61260. Dots represent each patients for each GSE, lines are the elliNAFLes centered to the mean (coloured squares) representing 95% interval confidence, and p the probability associated with the F-test of the analysis of variance along the axes of the first dimensions (α =0.05). GSE63067: Control, n= 6; patients with NAFL (NAFL), n= 2; patients with NASH (NASH) , n= 9. GSE17470: Control, n= 4; patients with NASH (NASH) , n= 7.

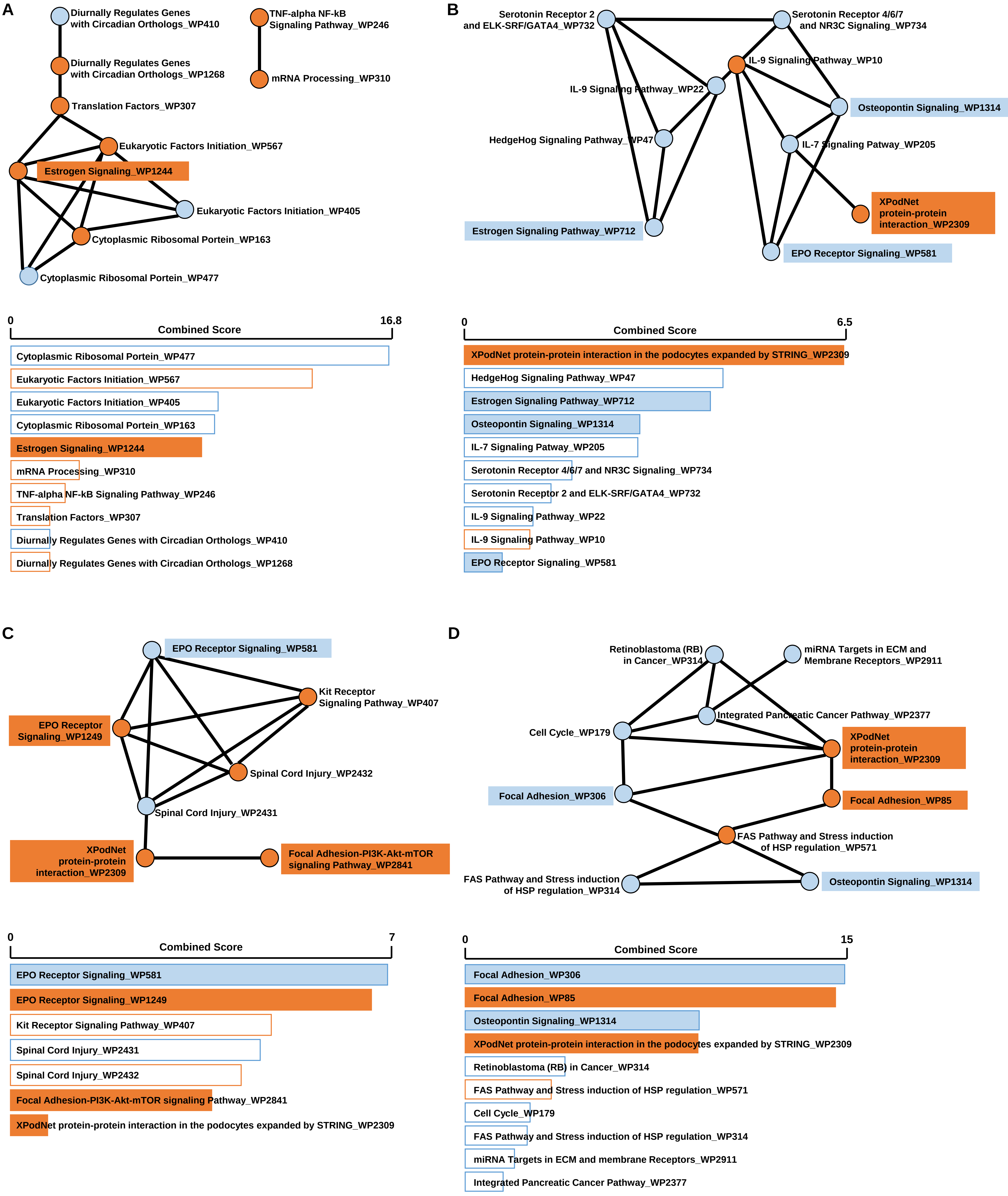

Supplementary Figure S3. Genes discriminating the 4 groups of patients from GSE48452 and GSE61260 belong to specific pathways. Among the 1549 genes selected, **(A)** 186 genes are specific to the healthy groups and belong to pathways implicated in circadian rhythm, general and hormonal metabolism; **(B)** 12 genes are specific to healthy obese patients and implicated also in circadian rhythm, hormonal metabolism, inflammation, extracellular matrix remodeling, nuclear receptors and transcription factors; **(C)** 9 genes are specific to obese patients with steatosis and implicated also in extracellular matrix remodeling, nuclear receptors and transcription factors; **(D)** 141 genes are specific to obese patients with NASH (nonalcoholic steatohepatitis) and implicated extracellular matrix remodeling, cancers, nuclear receptors and transcription factors. Boxes filled in orange and blue represent the common pathways between the four groups of patients. Pathway networks (top) were built using Enrichr website with Wikipathways (wikipathways.org) database from human (blue) and mouse (orange) databases. Data (bottom of each pathway network) are represented as horizontal bar graphs based on the combined score defined as the log of p-value from the Fisher exact test multiply by the z-score of the deviation from the expected rank. ECM: extracellular matrix pathways; EPO: erythropoietin pathway; FAS: apoptotic signaling pathway; HSP: heat shock protein; PodNet: podocyte network.

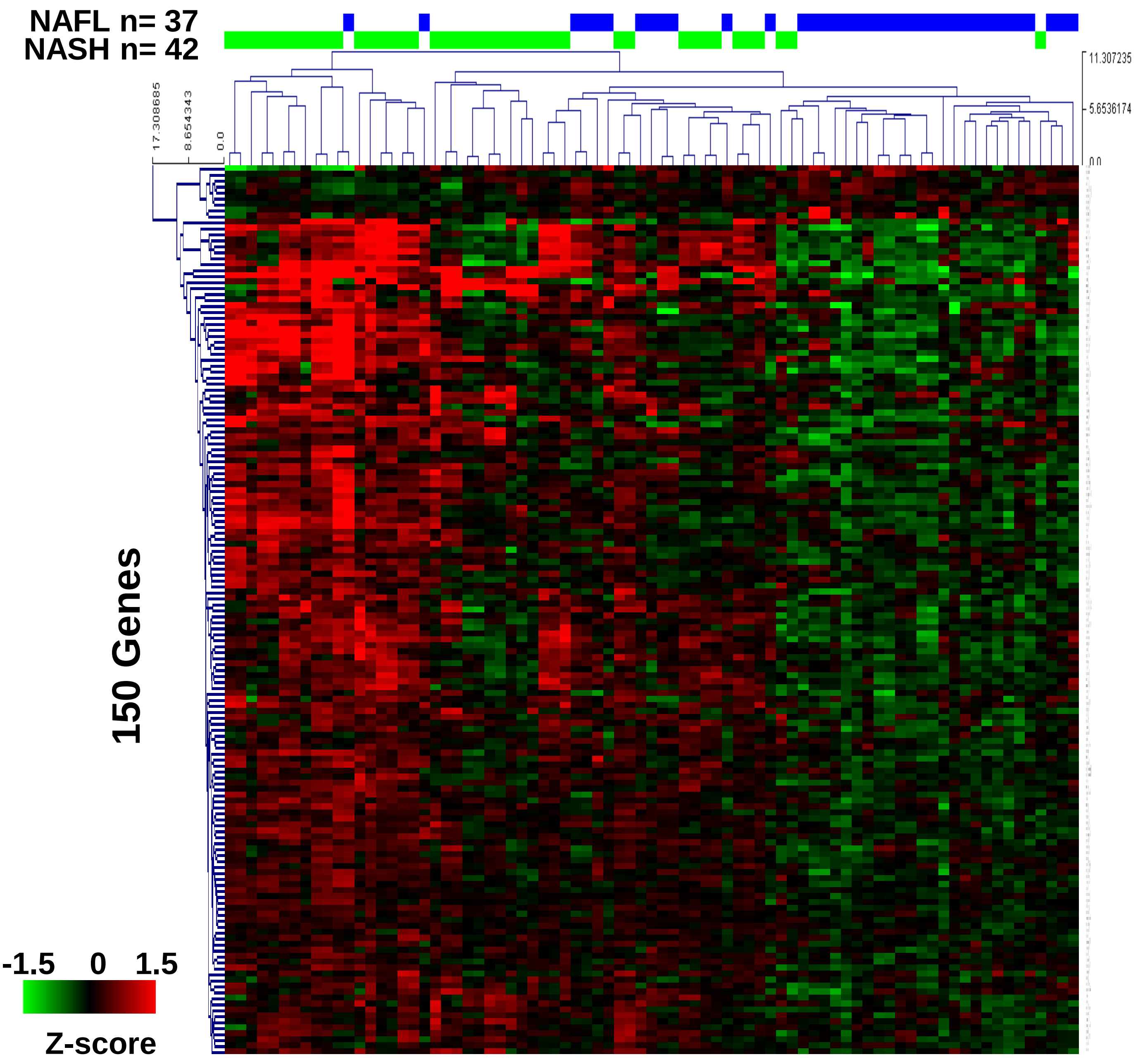

Supplementary Figure S4. Unsupervised analysis clustered 12 obese among 37 patients with steatosis (NAFL) with NASH (NASH, n=42). Heat map based on the 150 genes identified from NAFL (n= 9 genes) and NASH (n=141 genes) from both GSE48452 and GSE61260.

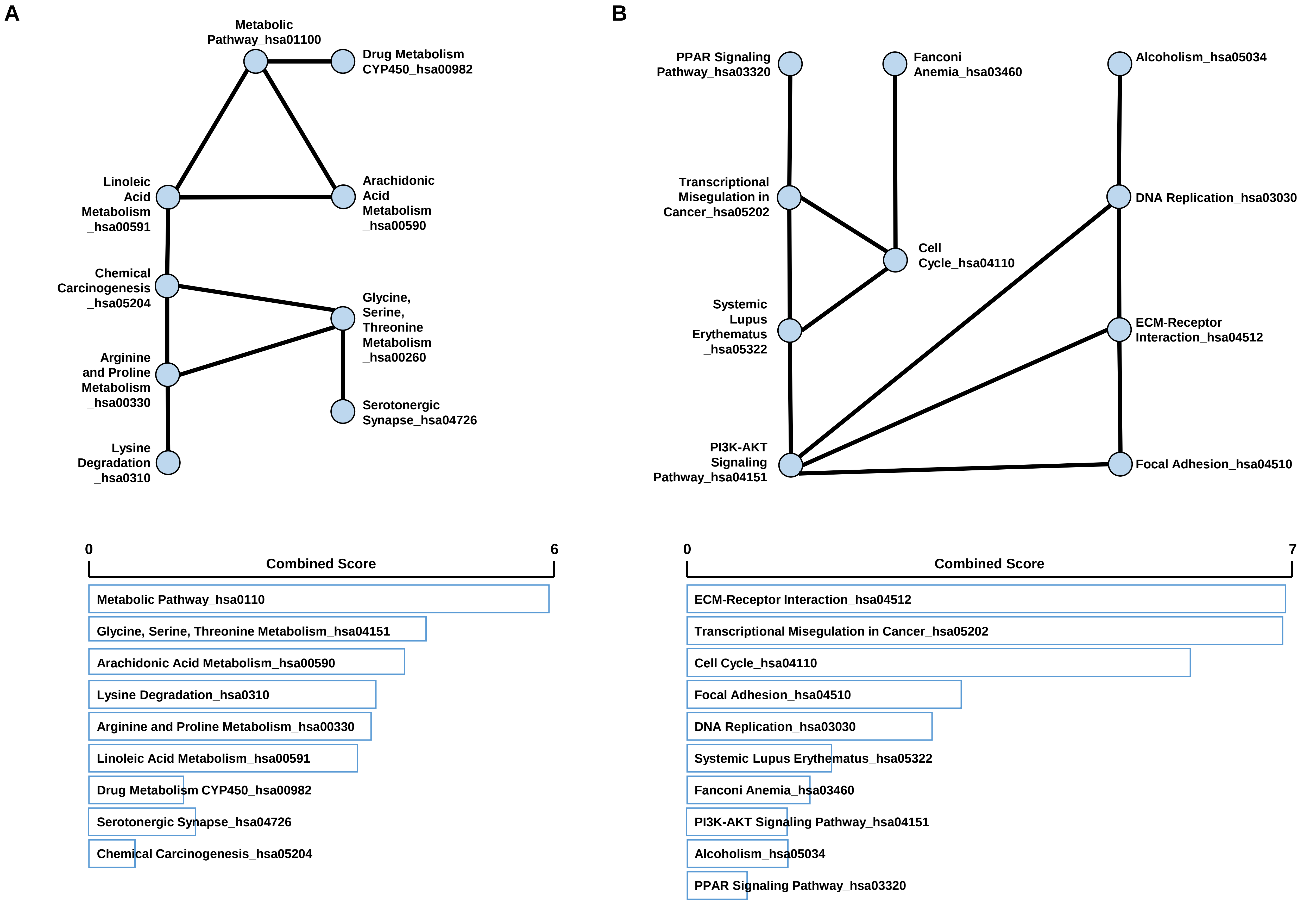

Supplementary Figure S5. Pathways underlying the 58 genes discriminating both subgroups of NAFL and NAFL mixed with NASH obese patients. (A) Pathways underlying the 4 genes down-regulated in MIX-NASH sub-groups (*i.e.* up-regulated in TRUE-Steatosis sub-group). **(B)** Pathways underlying the 54 genes up-regulated in MIX-NASH sub-groups. Pathway networks were built using Enrichr website and KEGG database. Data are represented as horizontal bar graphs based on the combined score defined as the log of p-value from the Fisher exact test multiply by the z-score of the deviation from the expected rank. Raw data are from both GSE48452 and GSE61260. ECM: extracellular matrix pathways; has: homo sapiens.

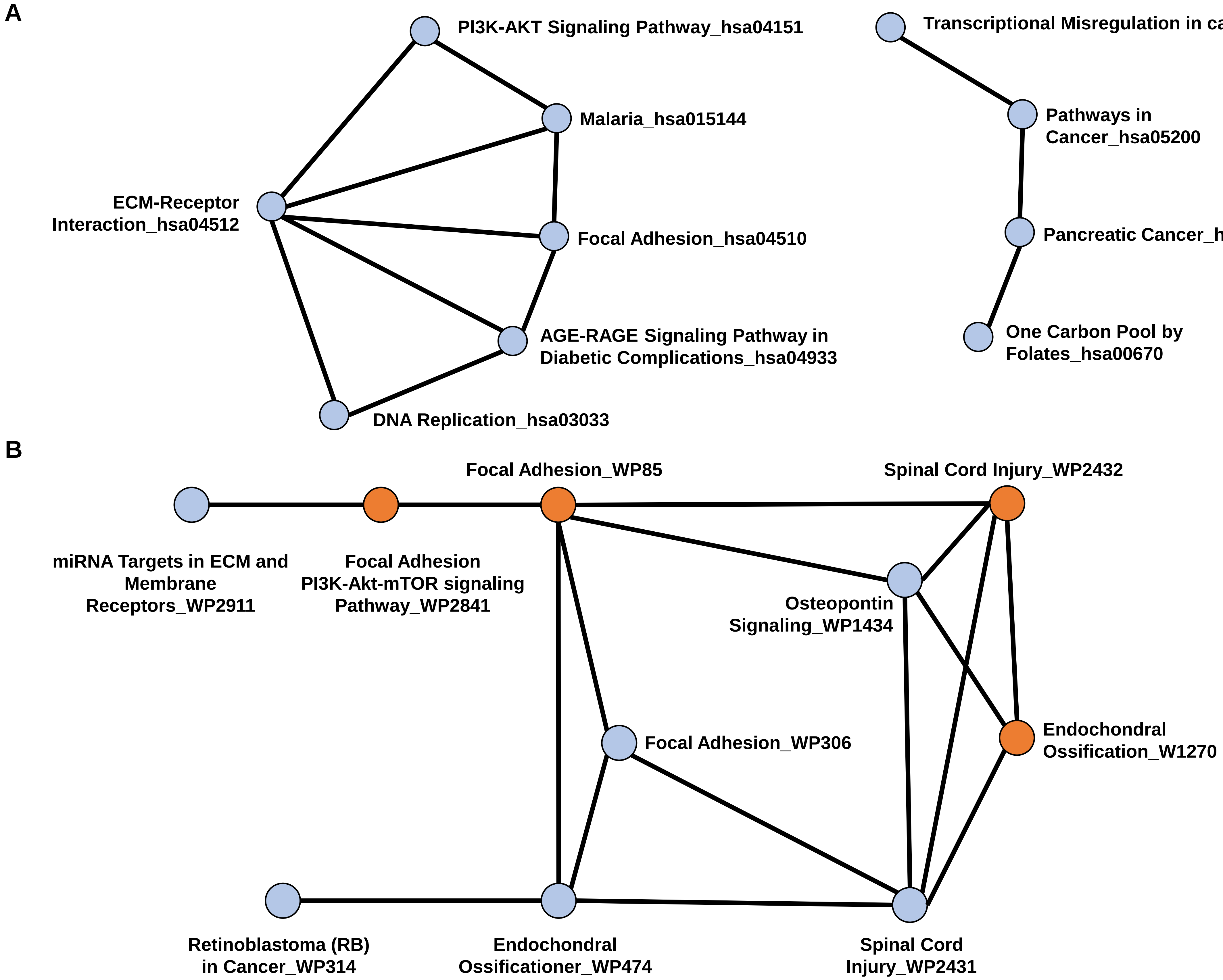

Supplementary Figure S6. The 14 genes in common between the 58 genes from NAFL patients and the 141 genes specific to NASH patients are associated to a poor prognosis. **(A)** Pathway networks were built using Enrichr website with **(A)** KEGG or **(B)** Wikipathways human ((blue dots) and mouse (orange dots) databases . Raw data are from both GSE48452 and GSE61260. **(E)** MMP-9 relative gene expression in each group of patients from both GSE48452 and GSE61260. Data are represented as boxplot. Threshold represents interval confidence 95% of the HP and identified 23 patients with high MMP-9 relative expression (red dots) and 19 patients with low MMP-9 relative expression (green dots). Control patients n=52; HO: healthy obese patients, n=38; NAFL obese patients, n= 35; NASH obese patients, n=41. ECM: extracellular matrix pathways; hsa: homo sapiens.

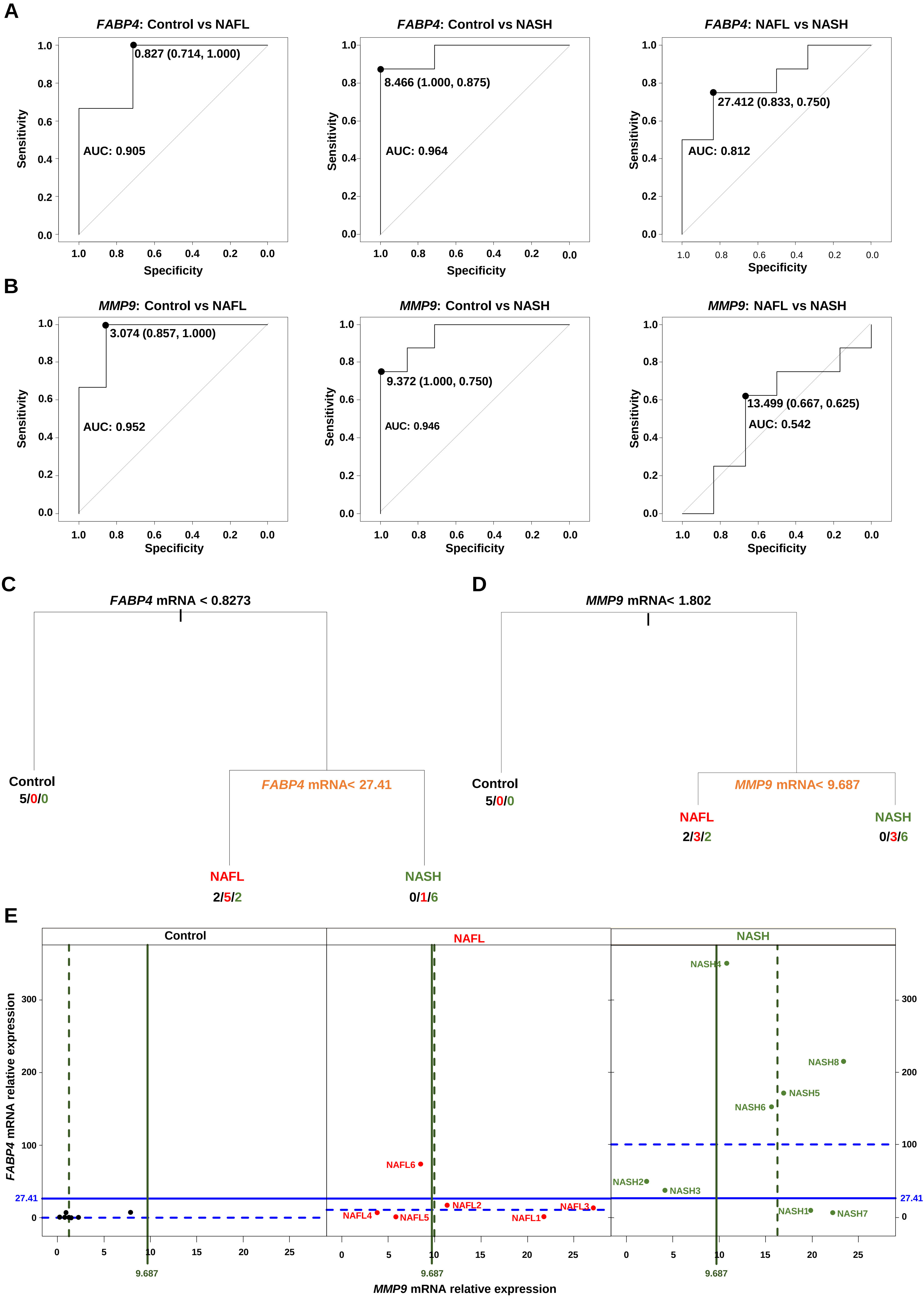

Supplementary Figure S7. *FABP4* and *MMP9* mRNA liver expression identified subgroups of NAFLD and NASH patients. Receiver operating characteristic (ROC) curve using **(A)** *FABP4* or **(B)** *MMP9* mRNA hepatic expression levels and comparing control versus NAFL (left panel), control versus NASH (middle panel) and NAFL versus NASH (right panel) patients. The Area Under the Curve (AUC) values are given, as well as the threshold, sensitivity, specificity at the optimal response cut-points (black dot). Comprehensive and regression trees (CART) of **(C)** *FABP4* and **(D)** *MMP9* mRNA hepatic expression levels comparing control (n=7), NAFL (n=6) and NASH (n=8) patients**. (E)** Plot of patients based on their *FABP4* (y-axis) or *MMP9* (x-axis) mRNA hepatic expression levels. Blue and green dash lines represents the median defined by the boxplots in Figure4B and Figure 5H, (respectively) and oranges lines the projection of the values determined by CART analysis in (C and D).
